# Supplementary material for: Geographic risk assessment of Batrachochytrium salamandrivorans invasion in Costa Rica as a means of informing emergence management and mitigation
Source: PLoS One. 2024 Dec 26;19(12):e0293779. doi: 10.1371/journal.pone.0293779 (PMC11670975; doi:10.1371/journal.pone.0293779)
Supplement: S1 Appendix — (DOCX) [file pone.0293779.s001.docx]

**Appendix I**

**Table I. Human visitation rate per annum for Costa Rican protected areas in 2018 (1).**
Note, visitation data was not found for all protected areas. Visitation numbers include both international and local visitors.

| **Name** | **Designation** | **Visitation** |
| --- | --- | --- |
| Manuel Antonio | National Park | 524,835 |
| Volcán Irazú | National Park | 422,947 |
| Marino Ballena | National Park | 165,875 |
| Tortuguero | National Park | 136,704 |
| Cahuita | National Park | 126,372 |
| Volcán Tenorio | National Park | 112,562 |
| Volcán Arenal | National Park | 111,286 |
| Rincon de la Vieja | National Park | 98,057 |
| Corcovado | National Park | 56,794 |
| Volcán Poás | National Park | 49,785 |
| Carara | National Park | 42,752 |
| Santa Rosa | National Park | 41,755 |
| Monumento Nacional Guayabo | National Monument | 30,985 |
| Tapantí - Macizo Cerro de la Muerte | National Park | 28,806 |
| Braulio Carrillo | National Park | 26,752 |
| Cipanci | National Wildlife Refuge | 21,553 |
| Chirripó | National Park | 21,079 |
| Grecia | Biological Reserve | 15,149 |
| Cabo Blanco | Absolute Natural Reserve | 12,086 |
| Ostional | National Wildlife Refuge | 9,578 |
| Palo Verde | National Park | 8,312 |
| Bahia Junquillal | National Wildlife Refuge | 7,537 |
| Barra Honda | National Park | 6,430 |
| Camaronal | National Wildlife Refuge | 2,491 |
| Monte Alto | Protected Zone | 2,085 |
| Lomas de Barbudal | Biological Reserve | 1,296 |
| Diria | National Park | 1,073 |
| La Cangreja | National Park | 1,002 |
| Internacional La Amistad | National Park | 744 |
| Marino las Baulas de Guanacaste | National Park | 721 |
| Golfito | National Wildlife Refuge | 721 |
| Barbilla | National Park | 517 |
| Penas Blancas | National Wildlife Refuge | 306 |
| Rio Macho | Biological Reserve | 197 |
| Guanacaste | National Park | 82 |
| Hitoy Cerere | Biological Reserve | 81 |
| Juan Castro Blanco | National Park | 61 |
| Gandoca Manzanillo | National Wildlife Refuge | 0 |
| Isla San lucas | National Wildlife Refuge | 0 |
| Playa Hermosa-Punta Mala | National Wildlife Refuge | 0 |
| Alberto Manuel Brenes | Biological Reserve | 0 |
| Los Quetzales | National Park | 0 |
| Volcán Turrialba | National Park | 0 |
| Cano Negro | National Wildlife Refuge | 0 |
| Cordillera Volcánica Central | Biological Reserve | 0 |
| Iguanita | National Wildlife Refuge | 0 |

**Table II. Salamander species, total land area, and protected land area in areas of high, medium, and low predicted *Bsal* suitability.**

| **Suitability** | **Total land area** (km^2^) | **Area protected** (km^2^) | **Area with four or more salamander sp.** (km^2^) | **Salamander species present** (*indicates endangered **indicates critically endangered status in Costa Rica) |
| --- | --- | --- | --- | --- |
| High | 78.62 | 72.70 | 16.90 | 16 species in total: *Bolitoglossa alvaradoi*, B. colonnea, B. diminuta, B. epimela, B. gracilis, B. pesrubra, B. robusta, B. subpalmata*, Oedipina alfaroi, O. cyclocauda, O. gracilis*, O. poelzi*, O. uniformis, Nototriton abscondens, & N. richardi* |
| Moderate | 11,585.39 | 6,503.18 | 9,153.83 | 36 species in total: *B. alvaradoi*, B. bramei*, B. cerroensis, B. colonnea, B. compacta*, B. diminuta, B. epimela*, B. gracilis, B. lignicolor, B. marmorea*, B. minutula*, B. nigrescens*, B. obscura, B. pesrubra, B. robusta, B. schizodactyla, B. sombra, B. sooyorum*, B. striatula, B. subpalmata*, B. tica*, N. abscondens, N. gamezi, N. guanacaste, N. major**, N. picadoi, N. richardi, O. alfaroi, O. carablanca*, O. collaris*, O. cyclocauda, O. gracilis*, O. grandis*, O. poelzi*, O. pseudouniformis*, & O. uniformis* |
| Low | 39,440.16 | 6,476.12 | 3,855.80 | 42 species in total: *B.alvaradoi*, B. bramei, B. cerroensis, B. colonnea, B. compacta*, B. epimela, B. gomezi, B. gracilis, B. lignicolor, B. marmorea*, B. minutula*, B. nigrescens*, B. obscura, B. pesrubra, B. robusta, B. schizodactyla. B. sombra, B. sooyorum*, B. striatula, B. subpalmata*, B. tica*, N. abscondens, N. gamezi, N. guanacaste, N. major**, N. picadoi, N. richardi, N. tapanti, O. alfaroi, O. alleni, O. altura*, O.carablanca*, O. collaris, O. cyclocauda, O. gracilis*, O. grandis*, O. pacificensis, O. paucidentata*, O. poelzi*, O. pseudouniformis*, O. savagei, O. uniformis* |

**Table III. Priority regions statistics.**This table lists the eight high priority areas suggested for monitoring. For each priority region, the total area in square kilometers that is considered highly and moderately suitable for *Bsal* is given. The average and range of suitability are listed (one indicates the habitat is highly suitable and zero indicates the habitat is completely unsuitable). Park visitation numbers are from 2018.

| Priority region | Area with high suitability (km^2^) | Area with medium suitability (km^2^) | *Bsal* suitability (0-1) | Salamander diversity and species | Salamander diversity in high suitability area | Visitation |
| --- | --- | --- | --- | --- | --- | --- |
| Parque Nacional Braulio Carrillo | 2.62 | 495.85 | Mean: 0.59  Range: 0.39-0.76 | 11  *Bolitoglossa alvaradoi,*  *Bolitoglossa colonnea,*  *Bolitoglossa robusta,*  *Bolitoglossa striatula,*  *Bolitoglossa subpalmata,*  *Nototriton abscondens,*  *Nototriton richardi,*  *Oedipina cyclocauda,*  *Oedipina gracilis,*  *Oedipina poelzi,*  *Oedipina uniformis* | 5  *Bolitoglossa robusta,*  *Bolitoglossa subpalmata,*  *Nototriton abscondens,*  *Oedipina poelzi,*  *Oedipina uniformis* | 26,752 |
| Parque Nacional Chirripó | 0.87 | 424.27 | Mean: 0.55  Range: 0.21-0.75 | 5  *Bolitoglossa cerroensis,*  *Bolitoglossa nigrescens,*  *Bolitoglossa pesrubra,*  *Bolitoglossa robusta,*  *Bolitoglossa sooyorum* | 1  *Bolitoglossa pesrubra* | 21,079 |
| Reserva de la Biosfera Cordillera Volcánica Central | 18.33 | 522.91 | Mean: 0.60  Range: 0.33-0.80 | 13  *Bolitoglossa alvaradoi,*  *Bolitoglossa colonnea,*  *Bolitoglossa robusta,*  *Bolitoglossa striatula,*  *Bolitoglossa subpalmata,*  *Nototriton abscondens,*  *Nototriton richardi,*  *Oedipina carablanca,*  *Oedipina cyclocauda,*  *Oedipina gracilis,*  *Oedipina poelzi,*  *Oedipina pseudouniformis,*  *Oedipina uniformis* | 7  *Bolitoglossa alvaradoi,*  *Bolitoglossa robusta,*  *Bolitoglossa subpalmata,*  *Nototriton abscondens,*  *Nototriton richardi,*  *Oedipina poelzi,*  *Oedipina uniformis* | 0 |
| Parque Nacional Volcán Irazú | 0 | 17.46 | Mean: 0.57  Range: 0.38-0.67 | 6  *Bolitoglossa robusta,*  *Bolitoglossa subpalmata,*  *Nototriton abscondens,*  *Oedipina poelzi,*  *Oedipina pseudouniformis,*  *Oedipina uniformis* | 0 | 422,947 |
| Parque Internacional La Amistad | 49.76 | 1339.14 | Mean: 0.56  Range: 0.20-0.79 | 13  *Bolitoglossa alvaradoi,*  *Bolitoglossa colonnea,*  *Bolitoglossa minutula,*  *Bolitoglossa pesrubra,*  *Bolitoglossa robusta,*  *Bolitoglossa schizodactyla,*  *Bolitoglossa sombra,*  *Bolitoglossa sooyorum,*  *Oedipina alfaroi,*  *Oedipina cyclocauda,*  *Oedipina gracilis,*  *Oedipina grandis,*  *Oedipina uniformis* | 5  *Bolitoglossa colonnea,*  *Bolitoglossa*  *pesrubra,*  *Oedipina alfaroi,*  *Oedipina cyclocauda,*  *Oedipina gracilis* | 744 |
| Parque Nacional Volcán Poás | 0 | 68.97 | Mean: 0.63  Range: 0.50-0.74 | 6  *Bolitoglossa alvaradoi,*  *Bolitoglossa robusta,*  *Bolitoglossa subpalmata,*  *Nototriton abscondens,*  *Oedipina poelzi,*  *Oedipina uniformis* | 0 | 49,785 |
| Parque Nacional Tapantí - Macizo Cerro de la Muerte | 3.49 | 477.52 | Mean: 0.58  Range: 0.31-0.78 | 16  *Bolitoglossa cerroensis,*  *Bolitoglossa colonnea,*  *Bolitoglossa diminuta,*  *Bolitoglossa epimela,*  *Bolitoglossa gracilis,*  *Bolitoglossa nigrescens,*  *Bolitoglossa obscura,*  *Bolitoglossa pesrubra,*  *Bolitoglossa robusta,*  *Bolitoglossa sooyorum,*  *Bolitoglossa striatula,*  *Bolitoglossa tica,*  *Nototriton picadoi,*  *Nototriton tapanti,*  *Oedipina collaris,*  *Oedipina gracilis* | 5  *Bolitoglossa diminuta,*  *Bolitoglossa epimela,*  *Bolitoglossa gracilis,*  *Bolitoglossa robusta,*  *Bolitoglossa pesrubra* | 28,806 |
| Parque Nacional Cahuita | 0 | 10.22 | Mean: 0.57  Range: 0.56-0.59 | 4  *Bolitoglossa colonnea,*  *Oedipina alfaroi,*  *Oedipina cyclocauda,*  *Oediopina gracilis* | 0 | 126,372 |

**Figure I. Salamander gamma diversity in protected and non-protected areas.**
Darker shades indicate higher gamma diversity (greater number of endemic species compared to all of Costa Rica). Protected areas are outlined and the number of visitors in 2018 is indicated by pattern.


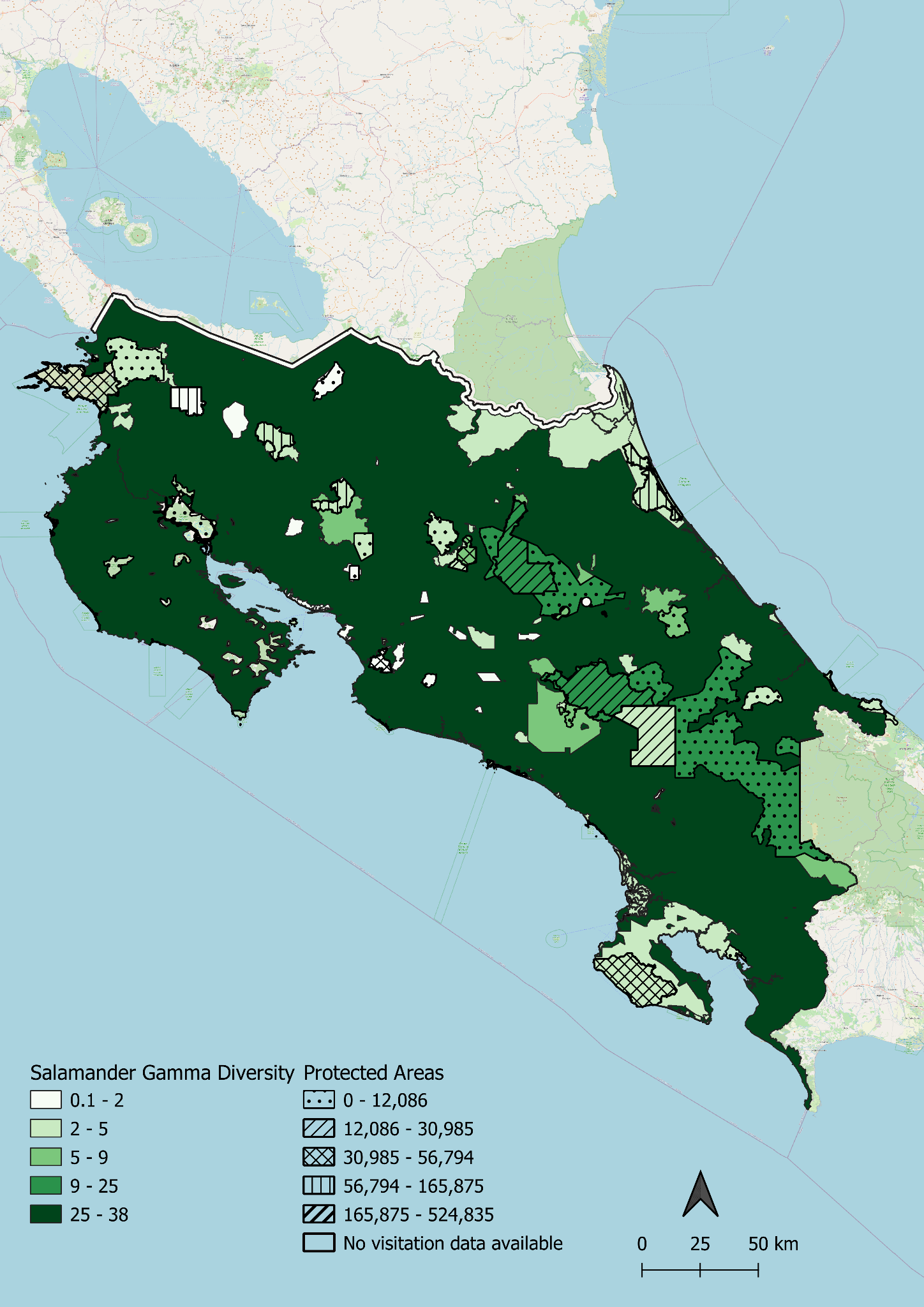


Reference

[1. Anuario Estadístico de Turismo 2018. San José, Costa Rica: Instituto Costarricense de Turismo; 2018.](https://www.zotero.org/google-docs/?V1gV2E)

**Model parameters and outputs for the model selected for the manuscript, hereinafter referred to as the “selected model”.**

Maxent version 3.4.1.

Regularized training gain is 1.942, training AUC is 0.946, unregularized training gain is 2.216.

Algorithm converged after 140 iterations (0 seconds).

The follow settings were used during the run:

31 presence records used for training.

10031 points used to determine the Maxent distribution (background points and presence points).

Environmental layers used (all continuous): maxent_bio_15_ASCII maxent_bio_18_ASCII maxent_bio_19_ASCII maxent_bio_2_ASCII maxent_bio_5_ASCII maxent_bio_7_ASCII

Regularization values: linear/quadratic/product: 0.247, categorical: 0.250, threshold: 1.690, hinge: 0.500

Feature types used: linear quadratic

jackknife: true

betamultiplier: 2.5

product: false

hinge: false

autorun: true

autofeature: false

**Table IV. Pearson’s correlation values for bioclimatic variables included in the selected model.** Cells with correlation values greater than an absolute value of 0.7 have been shaded with darker shading indicating greater correlation.

|  | **2** | **5** | **7** | **15** | **18** | **19** |
| --- | --- | --- | --- | --- | --- | --- |
| **2 Mean diurnal range** | 1.00 |  |  |  |  |  |
| **5 Max temperature of warmest month** | -0.25 | 1.00 |  |  |  |  |
| **7 Temperature annual range** | 0.92 | -0.25 | 1.00 |  |  |  |
| **15 Precipitation seasonality** | 0.19 | -0.34 | 0.29 | 1.00 |  |  |
| **18 Precipitation of warmest quarter** | -0.78 | 0.50 | -0.79 | -0.27 | 1.00 |  |
| **19 Precipitation of coldest quarter** | 0.52 | -0.47 | 0.65 | 0.81 | -0.60 | 1.00 |

**Table V. Analysis of variable contributions in the selected model.** To determine percent contribution, in each iteration of the training algorithm, the increase in regularized gain is added to the contribution of the corresponding variable or subtracted from it if the change to the absolute value of lambda is negative. For permutation importance, for each environmental variable in turn, the values of that variable on training presence and background data are randomly permuted. The model is reevaluated on the permuted data, and the resulting drop in training AUC is shown in the table, normalized to percentages. Variable contributions should be interpreted with caution when the predictor variables are correlated.

| **Variable** | **Percent contribution** | **Permutation importance** |
| --- | --- | --- |
| **2** | 56.3 | 64.3 |
| **18** | 20.9 | 1.2 |
| **7** | 20.3 | 28.2 |
| **5** | 2.2 | 6 |
| **19** | 0.4 | 0 |
| **15** | 0 | 0.3 |

**Figure II. Jackknife test of variable importance for the selected model.** Displays the variable importance of each environmental variable used in the selected model. Variable bioclimatic 2 contributes most to the model on its own.**
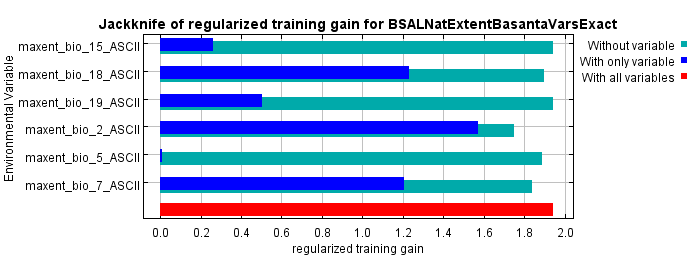
**

**Figure III. Analysis of omission/commission for the selected model.** Displays the omission rate, as calculated on the training presence records, and predicted area as a function of the cumulative threshold.


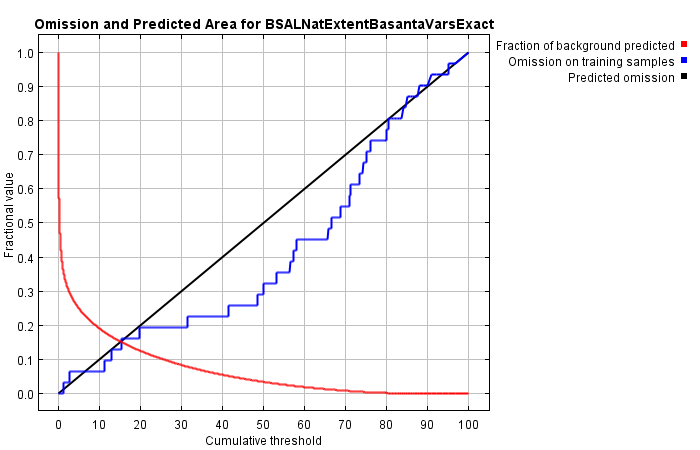


**Figure IV. The Receiver Operating Characteristic (ROC) curve for the selected model.**
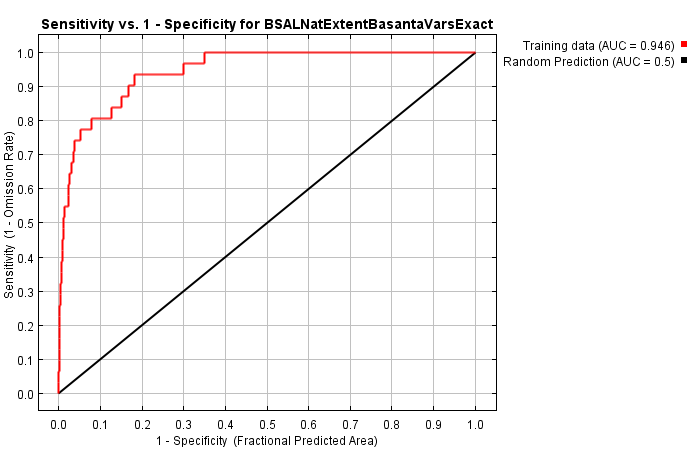


**Model parameters and outputs for the model using all 19 bioclimatic variables, hereinafter referred to as “all variables model”. This model was not ultimately selected by authors.**

Model with all 19 bioclimatic variables.

Maxent version 3.4.1.

The follow settings were used during the run:

Regularized training gain is 2.061, training AUC is 0.948, unregularized training gain is 2.376.

Algorithm converged after 340 iterations (0 seconds).

The follow settings were used during the run:

31 presence records used for training.

10030 points used to determine the Maxent distribution (background points and presence points).

Environmental layers used (all continuous): maxent_bio_10_ASCII maxent_bio_11_ASCII maxent_bio_12_ASCII maxent_bio_13_ASCII maxent_bio_14_ASCII maxent_bio_15_ASCII maxent_bio_16_ASCII maxent_bio_17_ASCII maxent_bio_18_ASCII maxent_bio_19_ASCII maxent_bio_1_ASCII maxent_bio_2_ASCII maxent_bio_3_ASCII maxent_bio_4_ASCII maxent_bio_5_ASCII maxent_bio_6_ASCII maxent_bio_7_ASCII maxent_bio_8_ASCII maxent_bio_9_ASCII

Regularization values: linear/quadratic/product: 0.247, categorical: 0.250, threshold: 1.690, hinge: 0.500

Feature types used: linear quadratic

jackknife: true

outputformat: logistic

betamultiplier: 2.5

product: false

hinge: false

autorun: true

autofeature: false

**Table VI. Pearson’s correlation values for bioclimatic variables included in the all variables model.** Cells with correlation values greater than an absolute value of 0.7 have been shaded with darker shading indicating greater correlation.

|  | **1** | **2** | **3** | **4** | **5** | **6** | **7** | **8** | **9** | **10** | **11** | **12** | **13** | **14** | **15** | **16** | **17** | **18** | **19** |
| --- | --- | --- | --- | --- | --- | --- | --- | --- | --- | --- | --- | --- | --- | --- | --- | --- | --- | --- | --- |
| **BIO1 = Annual Mean Temperature** | 1.00 |  |  |  |  |  |  |  |  |  |  |  |  |  |  |  |  |  |  |
| **BIO2 = Mean Diurnal Range (Mean of monthly (max temp - min temp))** | 0.50 | 1.00 |  |  |  |  |  |  |  |  |  |  |  |  |  |  |  |  |  |
| **BIO3 = Isothermality (BIO2/BIO7) (×100)** | 0.89 | 0.62 | 1.00 |  |  |  |  |  |  |  |  |  |  |  |  |  |  |  |  |
| **BIO4 = Temperature Seasonality (standard deviation ×100)** | 0.62 | 0.92 | 0.65 | 1.00 |  |  |  |  |  |  |  |  |  |  |  |  |  |  |  |
| **BIO5 = Max Temperature of Warmest Month** | -0.58 | -0.25 | -0.66 | -0.29 | 1.00 |  |  |  |  |  |  |  |  |  |  |  |  |  |  |
| **BIO6 = Min Temperature of Coldest Month** | 0.87 | 0.59 | 0.99 | 0.61 | -0.65 | 1.00 |  |  |  |  |  |  |  |  |  |  |  |  |  |
| **BIO7 = Temperature Annual Range (BIO5-BIO6)** | 0.61 | 0.92 | 0.63 | 0.99 | -0.25 | 0.59 | 1.00 |  |  |  |  |  |  |  |  |  |  |  |  |
| **BIO8 = Mean Temperature of Wettest Quarter** | 0.74 | 0.89 | 0.79 | 0.97 | -0.47 | 0.75 | 0.95 | 1.00 |  |  |  |  |  |  |  |  |  |  |  |
| **BIO9 = Mean Temperature of Driest Quarter** | 0.51 | 0.58 | 0.49 | 0.72 | -0.47 | 0.45 | 0.71 | 0.76 | 1.00 |  |  |  |  |  |  |  |  |  |  |
| **BIO10 = Mean Temperature of Warmest Quarter** | 0.33 | 0.35 | 0.37 | 0.43 | -0.39 | 0.34 | 0.44 | 0.46 | 0.67 | 1.00 |  |  |  |  |  |  |  |  |  |
| **BIO11 = Mean Temperature of Coldest Quarter** | 0.53 | 0.51 | 0.50 | 0.66 | -0.51 | 0.45 | 0.64 | 0.71 | 0.98 | 0.65 | 1.00 |  |  |  |  |  |  |  |  |
| **BIO12 = Annual Precipitation** | 0.22 | 0.33 | 0.25 | 0.38 | -0.27 | 0.23 | 0.40 | 0.38 | 0.61 | 0.97 | 0.57 | 1.00 |  |  |  |  |  |  |  |
| **BIO13 = Precipitation of Wettest Month** | -0.50 | -0.61 | -0.48 | -0.72 | 0.37 | -0.44 | -0.69 | -0.74 | -0.82 | -0.15 | -0.79 | -0.08 | 1.00 |  |  |  |  |  |  |
| **BIO14 = Precipitation of Driest Month** | 0.55 | 0.62 | 0.55 | 0.75 | -0.50 | 0.50 | 0.73 | 0.79 | 0.99 | 0.67 | 0.97 | 0.60 | -0.83 | 1.00 |  |  |  |  |  |
| **BIO15 = Precipitation Seasonality (Coefficient of Variation)** | 0.24 | 0.19 | 0.26 | 0.28 | -0.34 | 0.24 | 0.29 | 0.31 | 0.56 | 0.98 | 0.56 | 0.95 | 0.00 | 0.56 | 1.00 |  |  |  |  |
| **BIO16 = Precipitation of Wettest Quarter** | -0.40 | -0.48 | -0.35 | -0.61 | 0.29 | -0.31 | -0.57 | -0.62 | -0.78 | -0.05 | -0.76 | 0.01 | 0.97 | -0.77 | 0.07 | 1.00 |  |  |  |
| **BIO17 = Precipitation of Driest Quarter** | 0.10 | 0.04 | -0.11 | 0.23 | 0.05 | -0.16 | 0.21 | 0.20 | 0.48 | -0.12 | 0.49 | -0.12 | -0.61 | 0.42 | -0.14 | -0.75 | 1.00 |  |  |
| **BIO18 = Precipitation of Warmest Quarter** | -0.61 | -0.78 | -0.70 | -0.80 | 0.50 | -0.68 | -0.79 | -0.84 | -0.65 | -0.43 | -0.59 | -0.36 | 0.69 | -0.72 | -0.27 | 0.51 | 0.07 | 1.00 |  |
| **BIO19 = Precipitation of Coldest Quarter** | 0.46 | 0.52 | 0.47 | 0.65 | -0.47 | 0.42 | 0.65 | 0.68 | 0.93 | 0.88 | 0.91 | 0.83 | -0.58 | 0.93 | 0.81 | -0.51 | 0.25 | -0.60 | 1.00 |

**Table VII. Analysis of variable contributions for the all variables model.** To determine percent contribution, in each iteration of the training algorithm, the increase in regularized gain is added to the contribution of the corresponding variable or subtracted from it if the change to the absolute value of lambda is negative. For permutation importance, for each environmental variable in turn, the values of that variable on training presence and background data are randomly permuted. The model is reevaluated on the permuted data, and the resulting drop in training AUC is shown in the table, normalized to percentages. Variable contributions should be interpreted with caution when the predictor variables are correlated.

| **Variable** | **Percent contribution** | **Permutation importance** |
| --- | --- | --- |
| 12 | 30.2 | 0 |
| 2 | 24.7 | 16.5 |
| 7 | 11.9 | 77.4 |
| 17 | 9.7 | 0.3 |
| 18 | 8.2 | 3.2 |
| 13 | 7.3 | 0 |
| 3 | 2.2 | 0.9 |
| 5 | 1.6 | 1.5 |
| 4 | 1.4 | 0 |
| 6 | 1.2 | 0 |
| 14 | 0.9 | 0 |
| 16 | 0.8 | 0 |
| 8 | 0 | 0.3 |
| 9 | 0 | 0 |
| 1 | 0 | 0 |
| 19 | 0 | 0 |
| 15 | 0 | 0 |
| 11 | 0 | 0 |
| 10 | 0 | 0 |

**Figure V. Jackknife test of variable importance for the all variables model.** Displays the variable importance of each environmental variable used in the all variables model. Variable bioclimatic 2 contributes most to the model on its own.


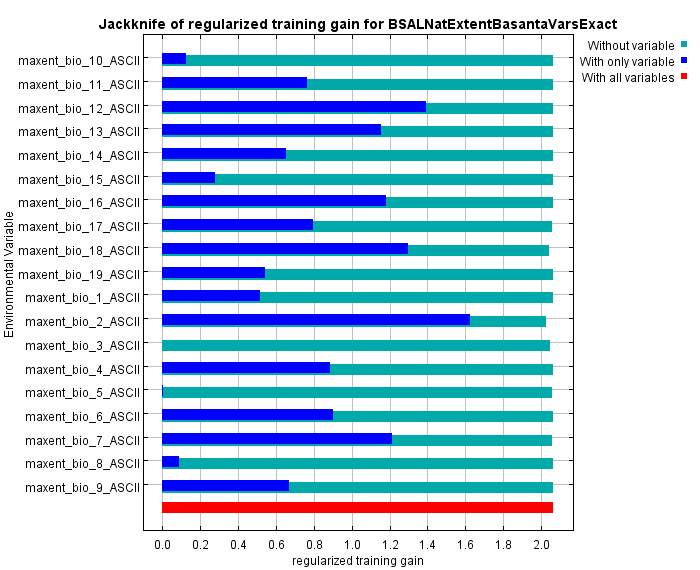


**Figure VI. Analysis of omission/commission for the all variables model.** Displays the omission rate, as calculated on the training presence records, and predicted area as a function of the cumulative threshold.


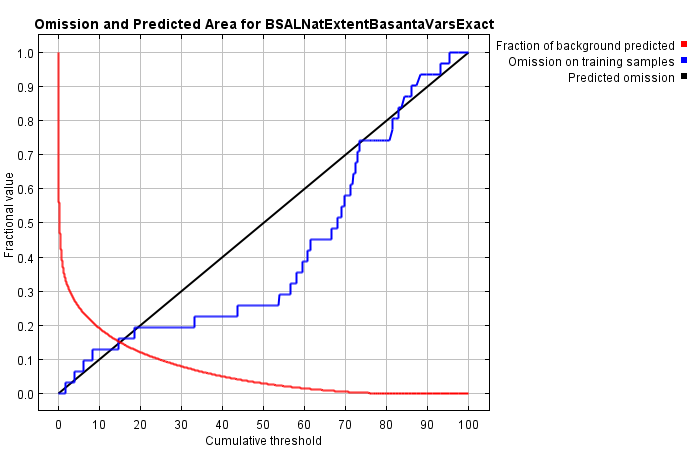


**Figure VII. The Receiver Operating Characteristic (ROC) curve all variables model.


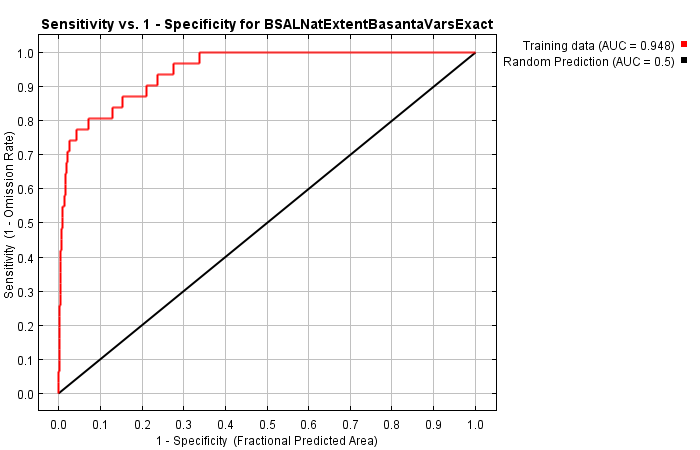
**

**Figure VIII. Predicted *Bsal* distribution in Costa Rica using 19 using the all variables model.** Warmer colors indicate higher suitability and cooler colors indicate lower suitability. Suitability ranges from 0-1. For more information on suitability, see full manuscript.

**
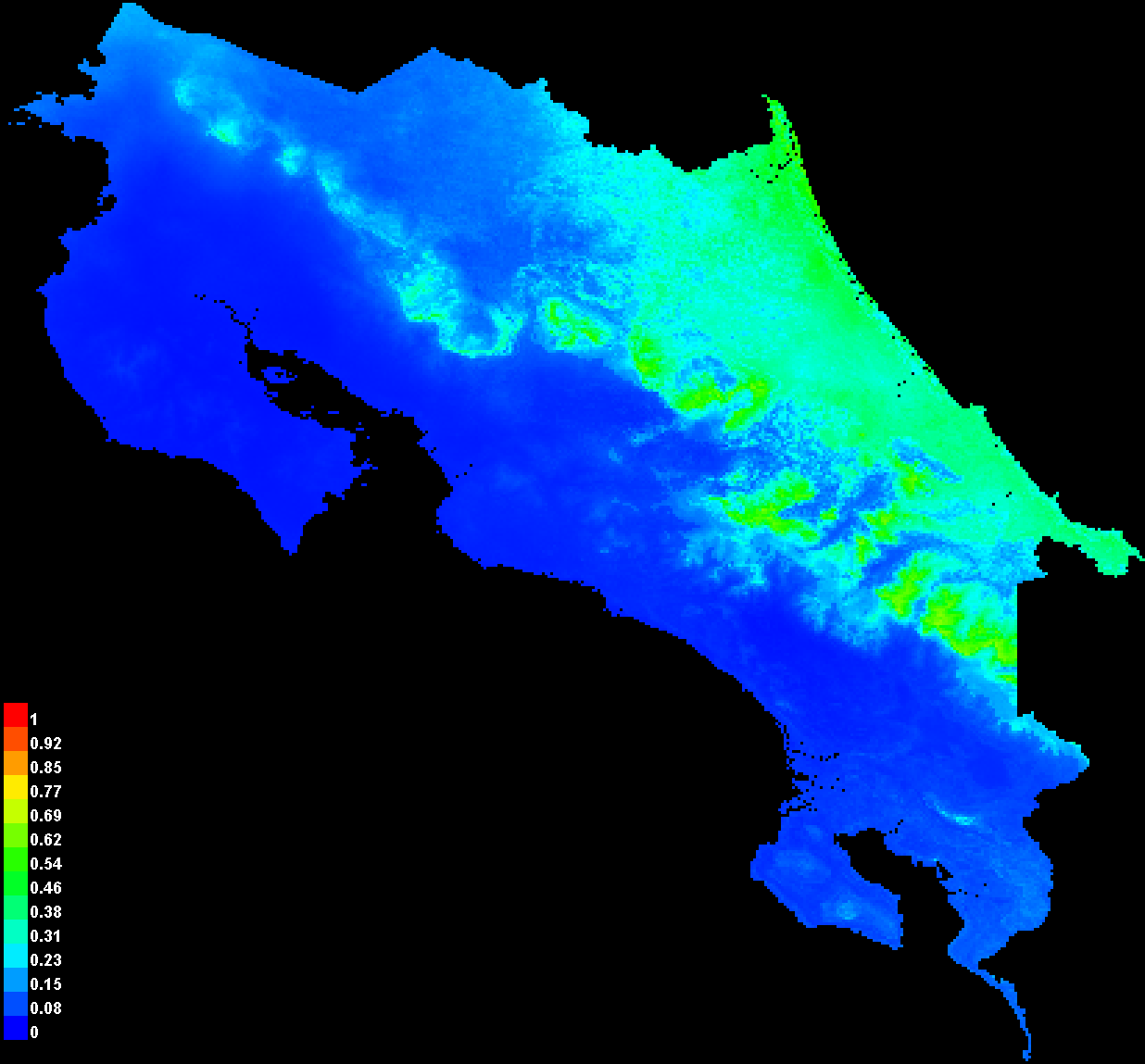
**

**Figure IX. Sensitivity. Difference between the selected model (Figure 4 in manuscript) and the all variables model (Figure VII).** Created by subtracting the suitability values of the selected model from the suitability values of the all variables model, which used uncorrelated variables. Positive values indicate greater suitability in the model chosen for the manuscript. Negative values indicate lower suitability in the model chosen for the manuscript. For more information on suitability, see the full manuscript.

**
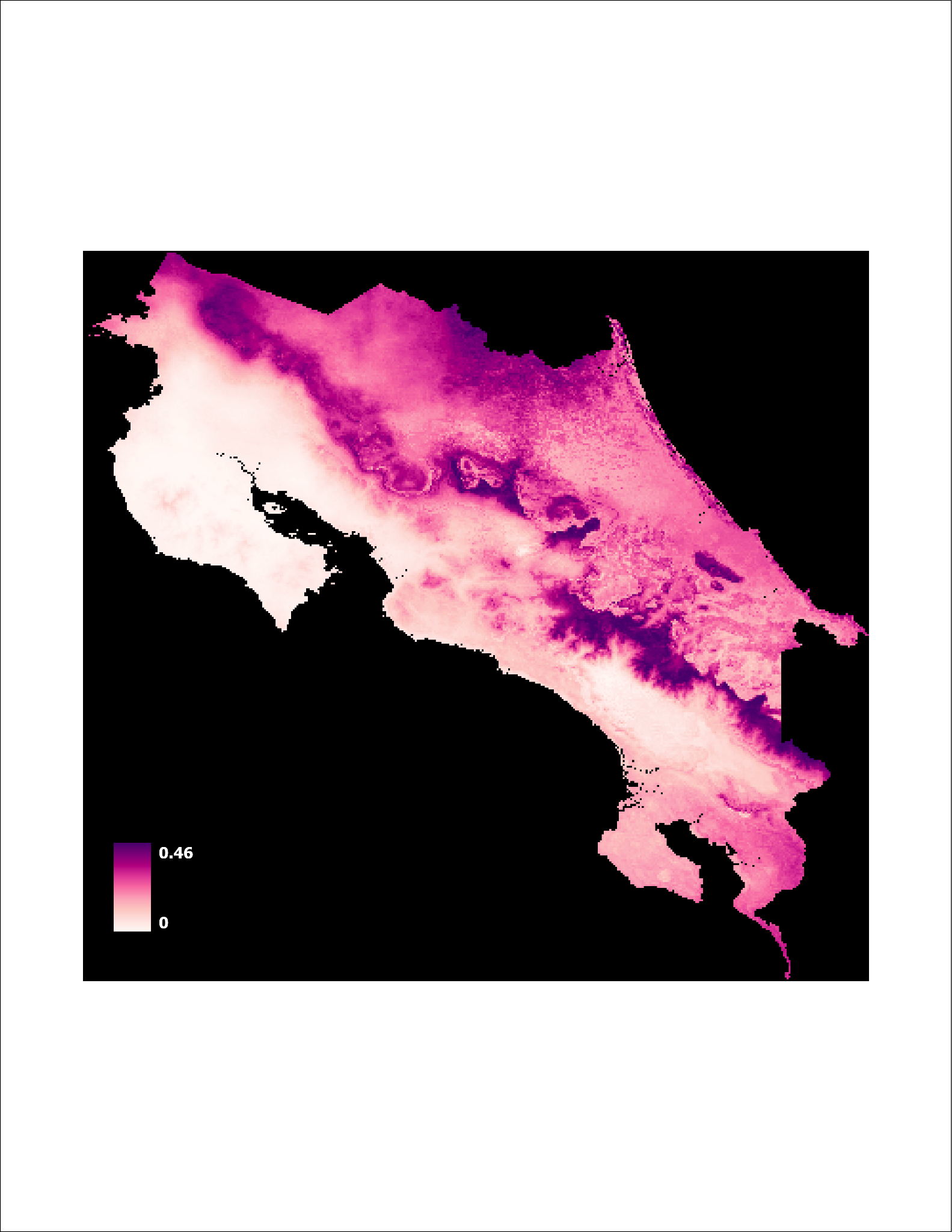
**

**Model parameters and outputs for the model using bioclimatic variables 1, 2, 5, 12, 13, and 17, hereinafter referred to as the “limited model”. This model was not ultimately selected by authors.**

Maxent version 3.4.1.

Regularized training gain is 1.914, training AUC is 0.948, unregularized training gain is 2.205.

Algorithm converged after 140 iterations (0 seconds).

The follow settings were used during the run:

31 presence records used for training.

10030 points used to determine the Maxent distribution (background points and presence points).

Environmental layers used (all continuous): maxent_bio_12_ASCII maxent_bio_13_ASCII maxent_bio_17_ASCII maxent_bio_1_ASCII maxent_bio_2_ASCII maxent_bio_5_ASCII

Regularization values: linear/quadratic/product: 0.247, categorical: 0.250, threshold: 1.690, hinge: 0.500

Feature types used: linear quadratic

responsecurves: true

jackknife: true

outputformat: logistic

betamultiplier: 2.5

product: false

hinge: false

autorun: true

autofeature: false

**Table VIII. Pearson’s correlation values for bioclimatic variables included in the limited model.**

|  | **1** | **2** | **5** | **12** | **13** | **17** |
| --- | --- | --- | --- | --- | --- | --- |
| **BIO1 = Annual Mean Temperature** | 1.00 |  |  |  |  |  |
| **BIO2 = Mean Diurnal Range (Mean of monthly (max temp - min temp))** | 0.50 | 1.00 |  |  |  |  |
| **BIO5 = Max Temperature of Warmest Month** | -0.58 | -0.25 | 1.00 |  |  |  |
| **BIO12 = Annual Precipitation** | 0.22 | 0.33 | -0.27 | 1.00 |  |  |
| **BIO13 = Precipitation of Wettest Month** | -0.50 | -0.61 | 0.37 | -0.08 | 1.00 |  |
| **BIO17 = Precipitation of Driest Quarter** | 0.10 | 0.04 | 0.05 | -0.12 | -0.61 | 1.00 |

**Table IX. Analysis of variable contributions in the limited model.**

| **Variable** | **Percent contribution** | **Permutation importance** |
| --- | --- | --- |
| 2 | 48.8 | 88.9 |
| 12 | 36.3 | 0 |
| 13 | 8.1 | 1.1 |
| 17 | 3.2 | 0 |
| 5 | 2.6 | 8.5 |
| 1 | 1 | 1.5 |

**Figure X. Jackknife test of variable importance for the limited model.** Displays the variable importance of each environmental variable used in the limited model. Variable bioclimatic 2 contributes most to the model on its own.


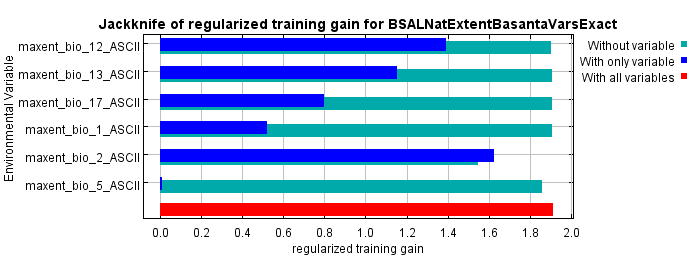


**Figure XI. Analysis of omission/commission for the limited model.** Displays the omission rate, as calculated on the training presence records, and predicted area as a function of the cumulative threshold.


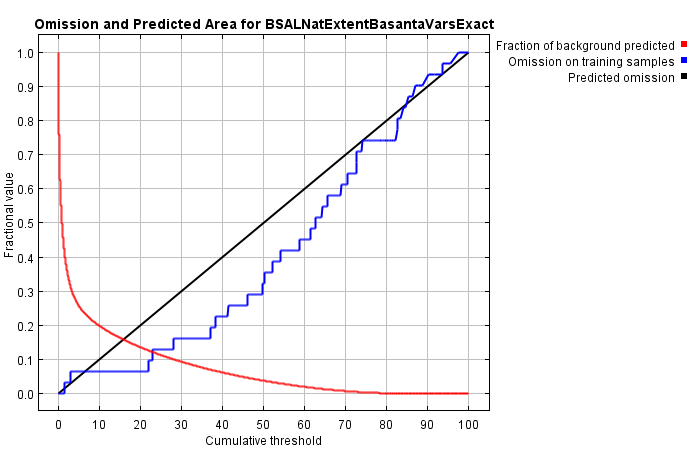


**Figure XII. The Receiver Operating Characteristic (ROC) curve for the limited model.**

**
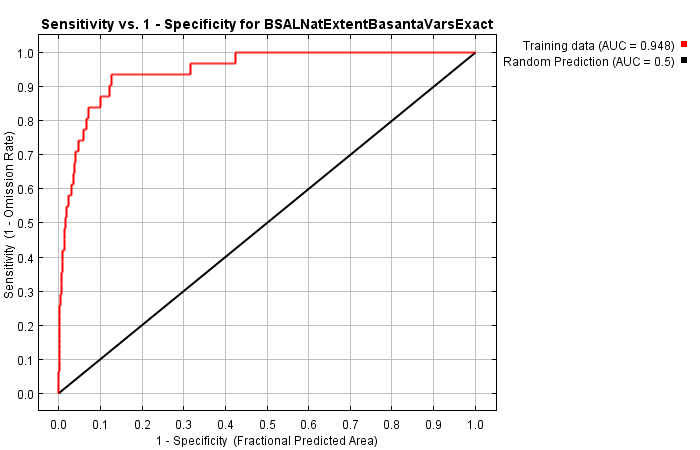
**

**Figure XIII. Predicted *Bsal* distribution in Costa Rica using the limited model.** Warmer colors indicate higher suitability and cooler colors indicate lower suitability. Suitability ranges from 0-1. For more information on suitability, see full manuscript.

**
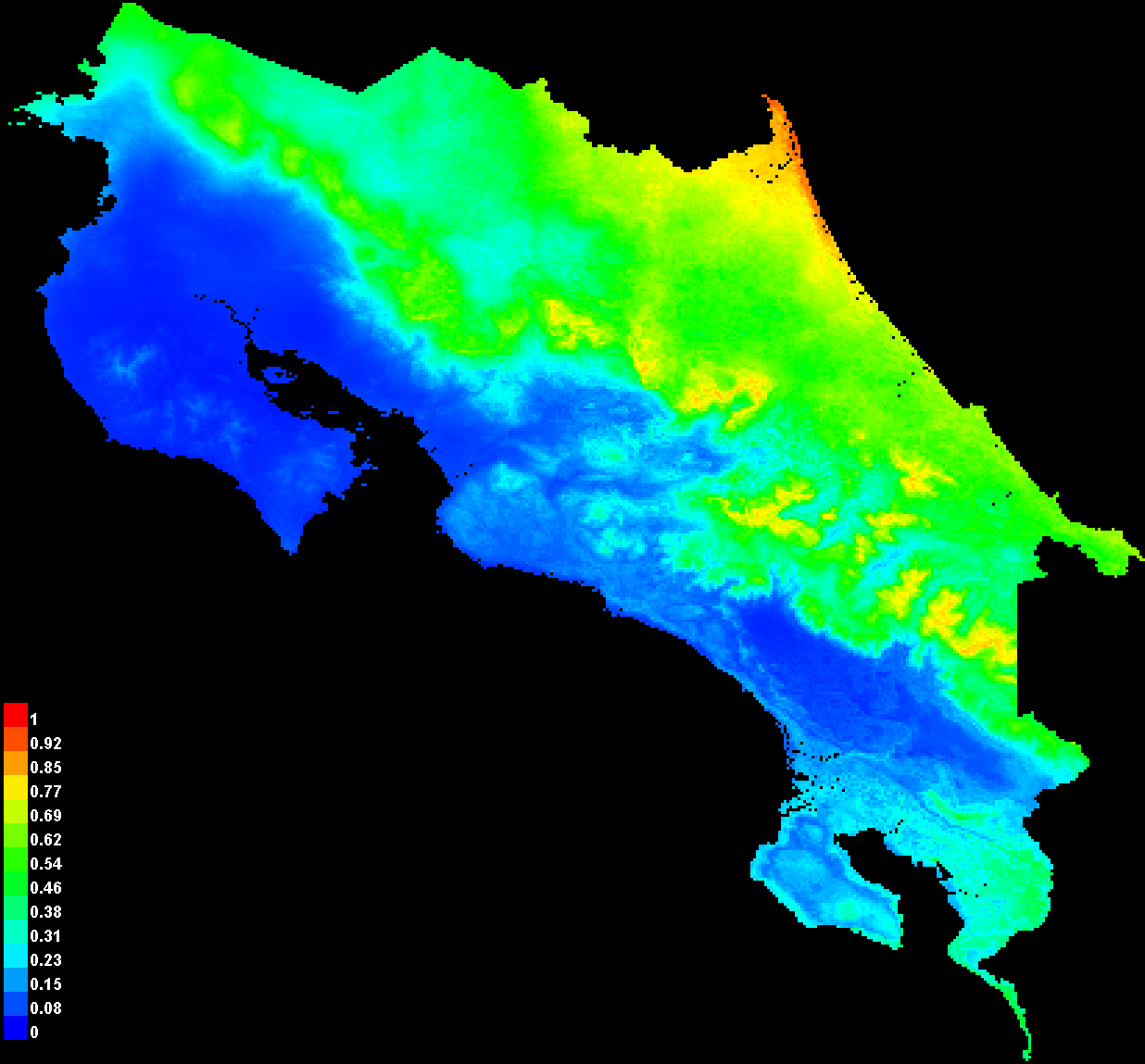
**

**Figure XIV. Sensitivity. Difference between the selected model (Figure 4 in manuscript) and the limited model, which used select, uncorrelated bioclimatic variables (Figure XII).** Created by subtracting the suitability values of the selected model from the suitability values of the limited model that used uncorrelated variables. Positive values indicate greater suitability in the model chosen for the manuscript. Negative values indicate lower suitability in the model chosen for the manuscript. For more information on suitability, see the full manuscript.

**
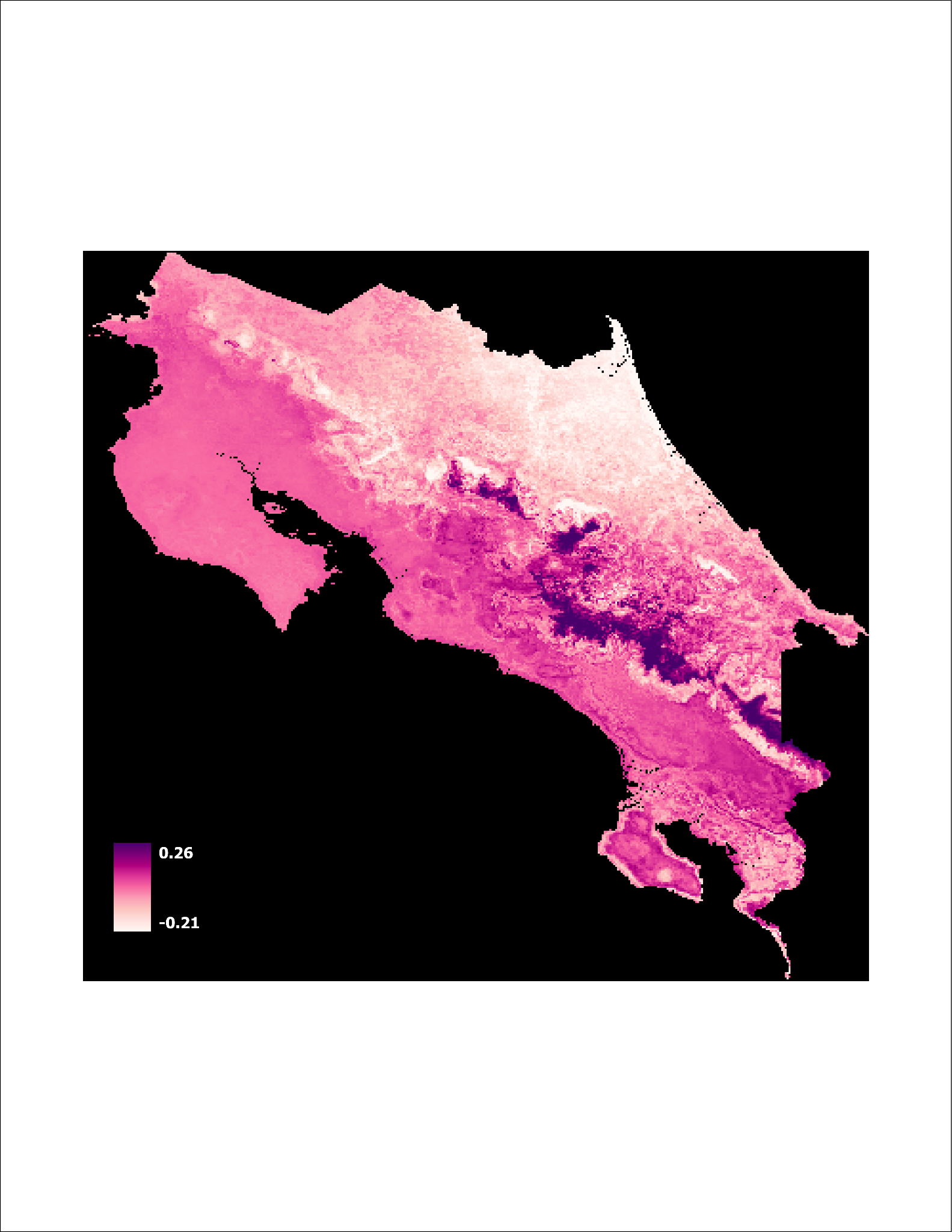
**
