# Supplementary material for: Geographic risk assessment of Batrachochytrium salamandrivorans invasion in Costa Rica as a means of informing emergence management and mitigation
Source: PLoS One. 2024 Dec 26;19(12):e0293779. doi: 10.1371/journal.pone.0293779 (PMC11670975; doi:10.1371/journal.pone.0293779)
Supplement: Attachment — Submitted filename: Response to Reviewers May 13th 2024.docx [file pone.0293779.s003.docx]

Dear Dr. Maidelyn Peregrin et al.

Thank you very much for your feedback regarding our manuscript “Geographic risk assessment of *Batrachochytrium salamandrivorans* invasion in Costa Rica as a means of informing emergence management and mitigation”.

To address the following statement **“Can you please clarify the specific origins of any basemaps, shapefiles, or map images utilized in constructing figures 2-7?”** we have edited figures 2-7 so that their basemaps are all sourced through Open Street Maps. Said basemaps may be accessed here: https://www.openstreetmap.org/#map=5/38.007/-95.844.

Additionally, all shape files used to create these figures are open source and can be accessed through Protected Planet: https://www.protectedplanet.net/country/CRI. All other information pertaining to the accessibility of the data used in this manuscript and to create our figures is provided in the “Additional Information” section of the manuscript editor platform. Please let us know if you have any further questions.

Best regards,

Henry C. Adams, Katherine E. Markham, Marguerite Madden, Matthew J. Gray, Federico Bolanos Vives, Gerardo Chaves, and Sonia M. Hernandez
