# Supplementary material for: Geographic risk assessment of Batrachochytrium salamandrivorans invasion in Costa Rica as a means of informing emergence management and mitigation
Source: PLoS One. 2024 Dec 26;19(12):e0293779. doi: 10.1371/journal.pone.0293779 (PMC11670975; doi:10.1371/journal.pone.0293779)
Supplement: Attachment — Submitted filename: Response to Reviewers.docx [file pone.0293779.s004.docx]

Dear Dr. Daniel Silva and reviewers,

Thank you very much for your time and the feedback you have provided regarding our manuscript “Geographic risk assessment of *Batrachochytrium salamandrivorans* invasion in Costa Rica as a means of informing emergence management and mitigation”. We are excited by the prospect of acceptance after addressing the latest reviewer comments, which we have addressed to the best of our abilities as outlined below. Please let us know if you have any further questions and thank you again for your time and expertise.

Best regards,

Henry C. Adams, Katherine E. Markham, Marguerite Madden, Matthew J. Gray, Federico Bolanos Vives, Gerardo Chaves, and Sonia M. Hernandez

**Reviewer #1 Comments**

Line 137: Is there any justification for using polygons instead of occurrence points? If so, it would be interesting to mention the advantages and limitations of this approach, as there may be differences at this study scale. Polygons tend to be more “optimistic” compared to occurrence points. – **Thank you for raising this issue. We have included a note about our estimates being optimistic in line 396 in the Discussion. We used polygons for modeling amphibian diversity because this is what IUCN provides and because TerrSet rasterizes polygons and creates biodiversity maps from these data. While we agree such methodology is imperfect, it is also not at all uncommon (i.e. Jenkins, Pimm, & Joppa 2013; Oliver et al., 2022; Wang et al., 2013). Our map of salamander biodiversity aligns with distribution as expected given the biological needs of this order in relation to elevation, temperature, precipitation, and so forth.**

**Jenkins, C. N., Pimm, S. L., & Joppa, L. N. (2013). Global patterns of terrestrial vertebrate diversity and conservation. Proceedings of the National Academy of Sciences, 110(28), E2602-E2610.**

**Oliver, P. M., Bower, D. S., McDonald, P. J., Kraus, F., Luedtke, J., Neam, K., ... & Richards, S. J. (2022). Melanesia holds the world’s most diverse and intact insular amphibian fauna. Communications biology, 5(1), 1182.**

**Wang, Y. C., Srivathsan, A., Feng, C. C., Salim, A., & Shekelle, M. (2013). Asian primate species richness correlates with rainfall. PLoS One, 8(1), e54995.**

Line 188 - Figure 1: As I understand it, the Range Restriction Index (RRI) was removed from the analyses, yet it still appears in Figure 1. The figure should be updated, or the index should be explained again in the methods. – **This figure has been updated.**

Line 197: You obtained data from only 46 out of 164 parks analyzed (less than 1/3). Did this require any treatment for overlapping suitability and visitation data? How did you handle or how does the analysis deal with these missing data? I noticed there is an area of high suitability for BSal but no visitor data in that region. – **If a park had no visitation data reported, it could only be identified as a high priority monitoring area if it contained high/very high *Bsal* suitability scores. We have clarified this in lines 193-195. Thank you for pointing out to us that this was not clearly stated.**

Line 253 - Table 1: It seems either the results text is too long, making the table unnecessary, or the text could be shorter, with the table serving as an additional way to interpret the results. My suggestion would be to move this table to the supplementary material. – **We have moved Table 1 to Appendix II (now Table II, Appendix II).**

Line 384: In my opinion, another limitation of the study is that you have visitation data for less than 1/3 of the parks, which also limits the areas and could be a recommendation. As the study mentions, this could be another way the fungus spreads. – **Excellent point. We've added statements to better highlight this limitation.**

**Reviewer #2 Comments**

Results: In the previous revision I mentioned that “The authors used different feature class combinations to construct the model, but, in the results section, it is not explained which was used as the best model, or how it was selected. Please add the information in the methods and results section.” The author’s mentioned that they used AICc to select the best model, but how were the AICc values of all models and the best model selected? Please add information of the best model, how was its parametrization, and a supplementary table with all model combinations and their AICc values, highlighting the best model selected.

**Upon reading Reviewer 2’s comments, we realize our previous description of our methods was too brief and unintentionally opaque. We now address specifically how we selected the best in the methods section. As Terrset does not provide AIC measures that can be compared between models, we thought it worthwhile to trust the validity of Basanta’s work in Mexico in identifying the environmental variables that should be included, but we also created two additional models: one using all 19 bioclimatic variables and one using only uncorrelated variables. Upon visually inspecting all three maps, we found the one ultimately included in this manuscript to be the most reasonable based on what is known about *Bsal*’s distribution and the climatic conditions under which it thrives, and based on our knowledge of Costa Rican biogeography.**

**For each model we considered, we have added model parameters, analysis of variable contributions, jackknife test of variable importance, analysis of omission and commission area, and the Receiver Operating Characteristic curve. For the two models that were not ultimately presented in the manuscript, we also included their predicted *Bsal* distribution map and a sensitivity analysis. This information has been added in the Appendices.**

I also have some minor comments:

77-80: Please add the reference Basanta et al. 2022
Basanta, M. D., Avila-Akerberg, V., Byrne, A. Q., Castellanos-Morales, G., Martínez, T. M. G., Maldonado-López, Y., ... & Rebollar, E. A. (2022). The fungal pathogen Batrachochytrium salamandrivorans is not detected in wild and captive amphibians from Mexico. PeerJ, 10, e14117. – **Thank you. This reference has been added.**

86-87: The first sentence of this paragraph is not connecting with the second. This paragraphs has two different ideas that are not connected. – **These statements have been edited for clarity.**

121: please check “although see” – **We have added a clause indicating this reference is to an argument against using smaller geographic extents to predict coverage.**

127- Due to the previous text didn’t mention the word “urodela/urodelan”, I suggest to change “species diversity of all urodelan” by “salamander diversity (all urodela species)” – **This change has been made**

151: Please add information and references of the “environmental variables”, How many and what resolutions were considered? **The environmental variables used in the model are explained further in the paragraph, specifically line 162-164. We have specified their resolution in line 164. We have added the Pearson correlation values for environmental variables in the Appendices.**

155: variables scoring greater than 0.7 of Pearson correlation were excluded, right? Please add a supplementary table with the Pearson correlation values. **Thank you for requesting the correlation matrix. Upon reviewing our models, we realized we had made a mistake and did not ultimately restrict variables with correlation scores greater than an absolute value of 0.7. We have removed this from the text, included that some of our variables are highly correlated (line 165-167), and have included tables with correlation values in the Appendix.**

156: please change “Bastanta and colleagues” by “Basanta and colleagues (2019)” – **This adjustment has been made.**

153&160: The authors mentioned that they used 34 presence points as Basanta and colleagues, but Basanta et al. used 32 presence points. Please add references or specify the sources of the extra two presence points. **Thank you for catching this mistake. We have updated line 154 to reflect that we used the same 31 presence points in *Bsal*’s natural range that Basanta and colleagues incorporated.**

161-164: Were these the variables selected in the “best model” ? Please add the information and check the comment below related to results.

**Yes, the bioclimatic factors listed in lines 162-164 are the environmental variables selected in the best model.**

168-169: here “Bsal” is without italic format. Please be consistent along the manuscript. – **Thank you for catching this mistake on our part. This correction has been made.**

299-300: How are the maximum temperatures in this area? How is considerate this “ideal thermal niche”? It is from experimental data previously published, or it is including field data of Bsal in Asia-Europe? Previous study found the presence of Bsal in ponds and streams with water temperatures between 20° - 25° C (Laking et al. 2017). – **During the warmest months of the year, maximum ambient temperatures in the northeast of Costa Rica range 25-30˚ and higher. When we discuss *Bsal’s* “ideal thermal niche” we are referencing both laboratory and *in situ* conditions, with appropriate references.**

Could the information of Figure 4 and Figure 7 be in a single figure? – **We feel that presenting the information in these two figures separately provides the reader with greater clarity of our manuscript's narrative.**
