# Supplementary material for: Geographic risk assessment of Batrachochytrium salamandrivorans invasion in Costa Rica as a means of informing emergence management and mitigation
Source: PLoS One. 2024 Dec 26;19(12):e0293779. doi: 10.1371/journal.pone.0293779 (PMC11670975; doi:10.1371/journal.pone.0293779)
Supplement: Attachment — Submitted filename: pone.0293779.docx [file pone.0293779.s005.docx]

Title: Geographic risk assessment of *Batrachochytrium salamandrivorans* invasion in Costa Rica as a means of informing emergence management and mitigation

Short Title: Risk assessment of *Bsal* in Costa Rica

Authors: Henry C. Adams ^1,2¶*^, Katherine E. Markham ^3,4¶^, Marguerite Madden ^3,4^, Matthew J. Gray ^5^, Federico Bolanos Vives ^6,7^, Gerardo Chaves ^7^, Sonia M. Hernandez ^2,8^

^1^ Urban Wildlife Institute, Lincoln Park Zoo Chicago, IL USA

^2^ Warnell School of Forestry, University of Georgia Athens, GA USA

^3^ Center for Geospatial Research, University of Georgia Athens, GA USA

^4^ Department of Geography, University of Georgia Athens, GA USA

^5^ Department of Forestry, Wildlife and Fisheries, University of Tennessee Knoxville, TN USA

^6^ Escuela de Biología, Universidad de Costa Rica, San Pedro, San José, Costa Rica

^7^ Centro de Investigaciones en Biodiversidad y Ecología Tropical (Museo de Zoología),
Universidad de Costa Rica, San Pedro, San José, Costa Rica

^8^ Southeastern Cooperative Wildlife Disease Study, College of Veterinary Medicine, University of Georgia Athens, GA USA

* Corresponding Author
E-mail: [hadams@lpzoo.org](mailto:hadams@lpzoo.org) (HCA)

¶ HCA and KEM are Joint Senior Authors

**Abstract**

Remotely-sensed risk assessments of emerging, invasive pathogens are key to targeted surveillance and outbreak responses. The recent emergence and spread of the fungal pathogen, *Batrachochytrium salamandrivorans* (*Bsal*), in Europe has negatively impacted multiple salamander species. Scholars and practitioners are increasingly concerned about the potential consequences of this lethal pathogen in the Americas, where salamander biodiversity is higher than anywhere else in the world. Although *Bsal* has not yet been detected in the Americas, certain countries have already proactively implemented monitoring and detection plans in order to identify areas of greatest concern and enable efficient contingency planning in the event of pathogen detection. To predict areas in Costa Rica with a high *Bsal* transmission risk, we employed ecological niche modeling combined with biodiversity and tourist visitation data to ascertain the specific risk to a country with world renowned biodiversity. Our findings indicate that approximately 23% of Costa Rica’s landmass provides suitable conditions for *Bsal*, posing a threat to 37 salamander species. The Central and Talamanca mountain ranges, in particular, have habitats predicted to be highly suitable for the pathogen. To facilitate monitoring and mitigation efforts, we identified eight specific protected areas that we believe are at the greatest risk due to a combination of high biodiversity, tourist visitation, and suitable habitat for *Bsal*. We advise regular monitoring utilizing remotely-sensed data and ecological niche modeling to effectively target *in-situ* surveillance and as places begin implementing educational efforts.

**Introduction**

Emergent infectious pathogens and their associated diseases pose an increasing threat to global wildlife biodiversity and ecosystem health (1). This threat calls for proactive management strategies that utilize pathogen invasion risk predictive models, targeted surveillance, and susceptibility assessment. Such strategies can better mitigate negative outcomes, reduce costs, and promote more efficient responses (2–4). In the case of *Batrachochytrium dendrobatidis* (*Bd*) and its associated chytridiomycosis in amphibians, decades passed between the pathogen’s initial emergence in the 1970s and the implementation of unified conservation action in 2005 and 2006 (2). By this time, regions of Central America and Australia had already lost upwards of 40% of amphibian biodiversity, due to *Bd* related chytridiomycosis, which has now been implicated in the decline of over 500 amphibian species worldwide (5–10). Potentially, as a result of lessons learned from the response to *Bd*’s emergence, a more rapid response was mobilized with the emergence of *Pseudogymnoascus destructans* (*Pd*), the causative agent of White-Nose Syndrome in bats. Management action plans designed to monitor and predict dispersal of the pathogen were drafted within four years of its emergence in 2006 in Howes Cave near Albany, New York (11–13). These proactive responses have allowed for the relatively rapid development of innovative *Pd* surveillance in North America, management strategies that accommodate for ecological variations (i.e., species, hibernation location, etc.), and anti-fungal treatment assessment and implementation (14–16).

As seen in the case of *Pd*, rapid proactive approaches to wildlife health management are crucial, especially in light of the recent emergence of the fungal pathogen, *Batrachochytrium salamandrivorans* (*Bsal*), the second chytrid fungus species known to parasitize amphibian hosts. Aptly dubbed the “salamander plague”, *Bsal* is a highly infectious saprophytic fungus with a wide thermal range (~5˚-25˚C) and presents a major global threat to salamanders (17–23). Emerging in Europe as early as 2004 after being introduced from East Asia, likely through the international pet trade, *Bsal* has caused significant declines in Central and Northern European salamander populations (7,17,18,21–25). The pathogen’s emergence in Europe has been met with highly proactive research and management measures. In 2016, regulations were implemented to limit the international movement and trade of salamanders in the United States and European Union. Continued susceptibility trials have illuminated taxonomic trends and nuances in the susceptibility of salamanders to *Bsal* and the ability of some anuran species to persist as subclinical, infectious hosts (19,20,25–27). Sweeping surveillance has been conducted in Europe, East Asia, Canada, Mexico, and the United States and has documented the expansion of *Bsal’s* non-native range, which now includes Belgium, Germany, The Netherlands, and Spain (2,3,18,24,28–33). In Spain, some *Bsal* detections have been made over 1,000 km away from previous reports. This highlights both the potential for *Bsal* to be moved great distances in non-native environments and the importance of continuous surveillance efforts (3,34,35). Fortunately, said detections were met with immediate collaborative efforts between scientists and regional authorities, including temporary wetland drainage and removal of *Bsal* positive individuals, that led to the successful containment of the pathogen (3).

The Americas are home to the world’s most diverse salamander communities and although *Bsal* has yet to be detected in the Western hemisphere, the execution and expansion of proactive conservation measures, similar to those taken in Spain, are of immense importance (32,36,37). Specifically, Costa Rica possesses the fifth most diverse salamander community in the world as well as a wealth of habitats potentially ecologically suitable for *Bsal* persistence (17,19,23,38,39). Much like *Bd*, *Bsal* is able to exist as an environmentally persistent zoospore, which, when in ideal conditions, may live independently of a host for up to 30 days (19). These zoospores may be moved through soil and water and can become attached to various clothing and equipment. As such, anthropogenic activities, be them recreational, commercial, or research focused, can facilitate the movement of *Bsal* to naïve environments, as exhibited in its initial emergence. Costa Rica has a thriving ecotourism industry with the majority of tourists originating from Europe and the United States annually (14% (~280,000) and 40% (~800,000) respectively) (40). It is believed that ecotourism and other anthropogenic activities exacerbated the emergence of *Bd* in Costa Rica during the 1980s and 1990s (7,41). With a large portion of annual visitors traveling from *Bsal’s* introduced range, a similar narrative could unfold with *Bsal* in Costa Rica and potentially impact other American salamander communities. This is why we believe it crucial to promote responsible recreation, proper research biosafety, and the development of management tools that may inform targeted *Bsal* surveillance. While surveillance for *Bsal* in Costa Rica has failed to detect the pathogen, such work, to our knowledge, has been limited to a single study that surveyed small geographic areas in the Cordillera de Talamanca and Central, highlighting the need for further surveillance efforts (39).

Ecological Niche Models (ENM, also referred to as Species Distribution Models) are valuable tools for understanding species’ habitat and therefore generating efficient and targeted surveillance efforts. ENM have been employed to evaluate the risk of invasion of *Bd* in Costa Rica, and *Bsal* in North America, Europe, and Central and South America (9,30,36,42–44). García-Rodríguez and colleagues recently generated *Bsal* ENM for Central and South America that identified multiple hotspots for *Bsal* introduction, mostly located in central and southern Mexico and Costa Rican mountain ranges (43,44). Despite the fact that the work by García-Rodríguez et al. (44) identifies sites where the diversity of salamanders and an optimal environment for *Bsal* converge, we believe that, in order to have a preventive effect on the transmission of the pathogen, we must consider the most probable sources of transmission and their relationship with the divers and susceptible areas. Indeed, recent work modeling *Bsal* spread in Europe has found that trail density is an important predictor of the pathogen’s presence (45). Our work advances our understanding of *Bsal* risk by modeling potential habitat using a smaller geographic extent than considered previously, potentially providing a more regionally specific risk assessment that accurately predicts occurrence (46) although see (47), which argues against using a smaller coverage.

We sought to identify areas of high risk in Costa Rica based on 1) *Bsal* ecological suitability, 2) salamander species diversity , and 3) the level of human visitation as a continuous source of possible pathogen introduction. Here, we developed an ENM similar to García-Rodríguez et al. (44) to identify areas ecologically suitable for *Bsal* and used data provided by the International Union for Conservation of Nature (IUCN) and the Costa Rican Institute of Tourism to evaluate amphibian distributions and human visitations, respectively. Our findings have the potential to provide a more nuanced spatial analysis of potential risk at a higher spatial resolution, leading to more precise and targeted monitoring efforts.

**Materials and methods**

To identify areas in Costa Rica at high risk of *Bsal* introduction, we considered salamander alpha biodiversity, human visitation, and habitat suitability to *Bsal* based on its current native range (18,23,48). High priority monitoring areas were identified based on how these three risk factors overlap. All modeling was done using TerrSet’s Habitat and Biodiversity Modeler (49).

Alpha diversity, or the total number of species within an area (roughly one km^2^), was calculated using a species’ extent data from the IUCN Red List (50,51). Alpha diversity is mapped on a continuous scale. To more easily communicate findings, we reclassified salamander alpha diversity into four qualitative categories: low (having no or one species of salamander), medium (two or three salamander sp.), high (four or five), and very high (six to eight). As monitoring and mitigation efforts are likely to be more feasible inside protected areas, we also calculated the gamma diversity, or regional species richness, of salamander species across all protected areas. Gamma diversity is useful when comparing an ecosystem or region's relative diversity to prioritize areas for conservation.

**Modeling potential *Bsal* habitat**

To identify potential suitable habitat for *Bsal* in Costa Rica, ecological niche modeling was conducted using *Bsal* occurrence records from within its endemic range. We used the maximum entropy model (Maxent), a correlative model that relates known species occurrences with environmental variables (52). Maxent models distribution using presence-only data, is highly accurate (53), and performs well with smaller sample sizes (54). Selecting only occurrence data from regions where *Bsal* is endemic, we trained our model using 31 presence points from previously published sources (23,48,55). We modeled our approach based on Basanta and colleagues’ (2019) predictions in Mexico; they trained Maxent species distribution models, and selected the one with the lowest lowest delta value of Akaike’s information criterion corrected for small sample sizes (AICc) (42). As the *Bsal* endemic occurrence records used in this study were identical to those used by Basanta and colleagues, we included the same environmental variables as Basanta and colleagues’ best predictive model: mean diurnal range, maximum temperature of the warmest month, temperature annual range, precipitation seasonality, precipitation of warmest quarter, and precipitation of coldest quarter (43,56). Our environmental data is at 0.5 by 0.5 degree resolution from the WorldClim bioclimatic dataset (56). While some variables are highly correlated (Table IV, Appendix I), Maxent is relatively stable when faced with correlated variables and internally deals with feature selection (57). We also created a model using all 19 environmental variables as well as a model using only variables that had an absolute value less than 0.7. As Terrset does not allow for comparison between models with AIC scores, we visually examined all three models and ultimately selected the model that used the same environmental variables as Basanta and colleagues (43). We chose this model because its produced distribution map and most significant predictive variables (i.e. temperature and precipitation) best aligned with our knowledge of Costa Rican bioclimates and the outputs and significant variables from previous studies predicting suitability for *Bsal* in Central America as well as *Bd*, which has similar ecological requirements to *Bsal* (9,38,43,44). Additionally, variables important to our model, such as temperature and precipitation, are highly influential on the pathogenicity of *Bsal* as well as the distribution of its amphibious hosts at coarser spatial scales, important factors to consider when assessing the risk of this pathogen’s introduction. (55,58,59). The two other models tested but ultimately not selected can be found in Appendix I.

Following Basanta and colleagues (2019), we used a parametrization of regularization multiplier (the degree to which predictions are fitted to training data) of 2.5 to obtain a more generalized output i.e., predictions are not overfitted to training data. We also used linear and quadratic feature class combinations so that the mean of each environmental variable in predicted occurrences matched that of observed occurrences and the variance in environmental variables for the predicted occurrences is constrained to that of observed occurrences. Model parameters and scores can be found in Appendix I. Maxent produces a map ranging from zero to one, with one indicating the most highly suitable habitat. We reclassified the suitability map into low (less than 0.5), medium (0.5-0.75), and high (over 0.75) suitability categories to simplify the interpretation of results.

High priority protected areas where monitoring efforts should be focused were identified as such if 1) *Bsal* suitability was high and caudata alpha diversity was high/very high or 2) *Bsal* suitability was moderate, caudata alpha diversity was high/very high, and reported annual visitation was above 40,000 individuals (the top 25% of human visitation for protected areas). If a park did not have visitation data available, it could only be identified as a high priority area for monitoring if it contained high or very high suitability for *Bsal*. An overview of the overall process for identifying *Bsal* monitoring areas in Costa Rica is shown in Figure 1.

**Figure 1. Process of identifying high priority monitoring areas.** Conceptual diagram showing the data, tools, and general processes used to identify areas highly suitable for *Bsal* that also have high levels of amphibian diversity. Circles indicate input data, outputs are outlined by squares, and final outputs are outlined by square dotted lines. Components of biodiversity modeling using amphibian data are shown with green gradient and components of habitat suitability mapping of *Bsal* are shown with blue gradient.

In addition to amphibian biodiversity and *Bsal* habitat suitability, we examined tourist visitation to protected areas using data provided by the Costa Rican Ecotourism Institute. Visitation data from 2018 were available for 46 out of 164 protected areas and included both national and international tourists (Figure 2). A list of the parks for which visitation data was available can be found in Table 1, Appendix I. Protected area data is from Protected Planet (60) and includes national parks, biological reserves, and United Nations Educational, Scientific and Cultural Organization (UNESCO) World Heritage Sites. By finding where high tourist visitation in parks overlaps with high/very high alpha diversity and high or moderate *Bsal* suitability, we further identified areas of high priority for pathogen surveillance and mitigative strategies.

**Figure 2. 2018 Human visitation in Costa Rica protected areas.** Protected areas include wildlife refuges, biological reserves, national parks, and world heritage sites. Protected areas are outlined in black. Where available, visitation numbers within protected areas for 2018 are indicated by color, with darker blue indicating more visitors. Insert map shows the geographic location of Costa Rica as a land bridge between North and South America.

**Results**

**Salamander alpha diversity**

Alpha diversity for salamanders was greatest in the central part of Costa Rica as well as along the Panamanian border and lowest in northwestern Costa Rica (Figure 3). Protected areas with high amphibian gamma diversity, or total biodiversity for Costa Rica, include Parque Internacional La Amistad, Parque Nacional Braulio Carrillo, Parque Nacional Tapantí - Macizo Cerro de la Muerte, Reserva Forestal Río Macho, and Reserva de la Biosfera Cordillera Volcánica Central (Table III, Appendix I).

**Figure 3. Costa Rican salamander alpha diversity.** These maps denote alpha diversity of salamanders in Costa Rica. Darker shades indicate higher alpha diversity. Alpha diversity was calculated by summing the total number of salamander species in each ~1 km by 1 km pixel.

**General *Bsal* suitability**

Based on our results, the Talamanca Mountain Range, the Central Mountain Range, and the Northern Caribbean coast of Costa Rica have the most suitable habitat conditions for *Bsal* (Figure 4). The highest habitat suitability scores observed across the Costa Rican landscape approached 0.80. Thus, of Costa Rica’s roughly 51,000 km^2^ landmass, we classified 77.33% as low (scores <0.50), 22.72% as medium (scores 0.5-0.75), and 0.15% as high suitability (scores >0.75) for *Bsal* (Figure 5).

**Figure 4. Predicted *Bsal* suitability in Costa Rica.** Higher suitability is indicated by reds and oranges and can be observed in the Cordillera Central and Cordillera de Talamanca mountain ranges, as well as along the northeastern Caribbean slope. Lower suitability is indicated by blues and greens.

**Figure 5.** **Predicted *Bsal* suitability categorized into high, medium, and low.** Protected areas are indicated by black diagonal lines. Insert map shows all of Costa Rica.

**Area of high *Bsal* suitability**

Eighteen percent of the land modeled to be highly suitable for *Bsal* is inhabited by four or more species of salamanders, and less than 5% of this land contains six or more species. In areas classified as highly suitable, there are a total of 15 species of salamanders, four of which are endangered (Table II, Appendix I). Ninety-two percent of this land falls within protected areas. The protected areas of Parque Internacional La Amistad, Parque Nacional Chirripó, Parque Nacional Tapantí - Macizo Cerro de la Muerte, Parque Nacional Braulio Carrillo, and the protected areas of the Central Volcanic Mountain Range Reserva de la Biosfera Cordillera Volcánica Central all have habitats modeled to be highly suitable (Figure 5). High *Bsal* suitability overlaps with high alpha salamander biodiversity in Reserva de la Biosfera Cordillera Volcánica Central, Parque Nacional Braulio Carrillo, and in Parque Internacional La Amistad along the border of the protected area Río Banano (Figure 6).

**Figure 6. Areas of overlap between predicted moderate and high *Bsal* suitability and high and very high salamander diversity.** Areas of both *Bsal* moderate suitability and high/very high salamander diversity are shown in yellow. Areas highly suitable for *Bsal* with high/very high salamander diversity are shown in red. Protected areas are outlined and the number of visitors in 2018 is indicated by the pattern in the legend.

**Area of moderate *Bsal* suitability**

Of the area considered moderately suitable for *Bsal*, 18% contains between six and eight species of salamanders and 43% contain four or five species. In total, 37 of the 44 salamander species included in this study are found in areas of moderate ecological suitability for *Bsal* (Table II, Appendix I)*.* These 37 species include the critically endangered *Nototriton major* and 13 other salamanders listed as endangered by the IUCN. Just over 56% of land modeled to be moderately suitable for *Bsal* is within protected areas (Table II & III, Appendix I), including parks with considerable tourist visitation, such as Parque Nacional Volcán Irazú, Parque Nacional Tortuguero, Parque Nacional Cahuita, Parque Nacional Volcán Tenorio and Parque Nacional Volcán Arenal, which all had over 100,000 visitors in 2018 (40).

**Identifying priority areas for surveillance and other management actions**

To identify parks of priority for *Bsal* surveillance, we first found areas of overlap between high *Bsal* suitability and high/very high salamander diversity (i.e. four or more species). Parque Nacional Braulio Carrillo, Parque Internacional La Amistad, Parque Nacional Tapantí - Macizo Cerro de la Muerte, Parque Nacional Chirripó, Reserva de la Biosfera Cordillera Volcánica Central have habitat that is both predicted to be highly suitable for *Bsal* and contains high/very high salamander diversity. We also considered areas that contain an overlap of high/very high salamander alpha diversity, moderate pathogen habitat suitability, and high human visitation in 2018 (>40,000 visitors). These areas include Parque Nacional Volcán Irazú, one of the most visited parks in 2018 with 400,000 visitors, Parque Nacional Cahuita (~126,000), as well as Parque Nacional Volcán Poás (~50,000 visitors) (Figure 7). See Table III, Appendix I for more details.

**Figure 7.** **Areas considered high priority for monitoring for *Bsal* introduction.** Protected priority areas outlined in black and identified by name overlaid on Ecological Niche Model results for *Bsal* suitability. Areas of warmer colors (reds and oranges) indicate high *Bsal* suitability, whereas cooler colors indicate lower suitability.

**Discussion**

***Bsal* Suitability**

Costa Rica’s mountain ranges, specifically the Cordillera Central and Cordillera de Talamanca, are predicted to be the country’s most ecologically suitable areas for *Bsal*. This is in agreement with habitat suitability modeling completed at the scale of the entire American tropics (44). Additionally, suitability is high in the Caribbean tropical forests of northeastern Costa Rica (Figure 4). This was not anticipated because this region’s maximum temperatures during the warmest months of the year frequently range outside of *Bsal’s* ideal thermal niche of 5-25˚C (56). Using ecological niche modeling, Basanta et al. (43) found high ecological suitability for *Bsal* in areas of Mexico bioclimatically similar to the northeastern coast of Costa Rica: tropical wet and rainforests with high ambient temperatures and high levels of precipitation. Basanta’s model and our model both found precipitation and temperature to be highly predictive of *Bsal* ecological suitability. It is possible that the high precipitation in the northeast of Costa Rica and the aforementioned regions of Mexico offsets the impact of ambient temperature on *Bsal* suitability in our model. It is also important to consider the role microhabitats play in creating thermal buffers in tropical regions. Tropical microhabitats have been found to be 1-2˚C cooler than the macrohabitat’s mean ambient temperature and upwards of 3.5˚C cooler than the macrohabitat’s maximum ambient temperature (61,62). The availability of these microhabitats as potential thermal refuges for *Bsal* as well as its hosts should be considered in the future development of fine scale occupancy models and execution of pathogen surveillance measures.

**Costa Rican salamander distribution & *Bsal***

Over 20% of Costa Rica’s landmass is ecologically suitable for *Bsal*. Though many areas identified as highly suitable for *Bsal* are within or adjacent to protected areas with limited public access and relatively low salamander alpha diversity, they could provide important footholds for *Bsal* introduction in Costa Rica. Additionally, many of the salamander species in areas highly suitable to *Bsal* are highly endemic to those regions (38). In the event of *Bsal* introduction, such endemism elevates the risk of not only severe population decline but also extirpation. While the transmission of *Bsal* is positively related to host density, it has been shown that outbreaks may still occur in environments with low host densities, provided appropriate environmental and host population connectivity (34,63). Understanding the densities of and connectivity between potential host populations will be crucial for creating more accurate mitigative tools.

Our results must be interpreted with some degree of uncertainty, as the susceptibility of specific Costa Rican salamander species to *Bsal* has yet to be determined. Such knowledge would add specificity to targeted surveillance efforts. This highlights the urgent need for susceptibility trials to be conducted with Costa Rican salamanders, especially those belonging to the genus *Bolitoglossa*. With at least 132 species existing only in the American tropics, this is the most diverse *Plethodontidae* genus and accounts for the majority of Costa Rica’s salamander biodiversity (38,64). *Plethodontidae* species, which includes all Costa Rican salamanders, are known to experience variable susceptibility to the fungus, with some being highly susceptible to infection, clinical disease, and mortality (18,20,65). As such, the large amount of overlap in *Bsal* ecological suitability and vulnerable salamander biodiversity could present a significant risk to the Costa Rican salamander community. Understanding *Plethodontidae*’s susceptibility to *Bsal* could greatly help predict and formulate management action plans for the fungus not only in Costa Rica but throughout the Americas (44).

Amphibian communities in Costa Rica have already been significantly impacted by *Bd* related epizootics, with some regions experiencing upwards of a 40% decline in species (9). Though *Bd* surveillance in Costa Rican salamanders has been limited, the pathogen is presumed to be endemic within their populations, given the well documented impact of *Bd* on anurans in Costa Rica and *Bolitoglossine* salamanders in Mexico and Guatemala (41,66). Eastern newts co-infected with *Bd* and *Bsal*, showed an increased susceptibility to *Bsal* and a greater downregulated immune response than newts infected with only *Bsal* (67). In the case of *Bsal* introduction to Costa Rica, it is believed that the combined impacts of both chytrid fungi on host populations would be detrimental. Based on the predictions of our ENM and a similar ENM produced by Puschendorf and colleagues, many areas ecologically suitable for *Bsal* in Costa Rica are also ecologically suitable for *Bd* (9). Such overlap exists in regions that have already experienced significant *Bd*-related amphibian population declines, specifically the Monteverde region and the Las Tablas Protected Zone. Monitoring for *Bsal* in these areas, including a handful of our identified areas of priority outlined in Table II, could be crucial to conserving these already degraded amphibian communities.

**Suggested management actions**

*Bsal* has not yet been detected in Costa Rica, but regular monitoring is needed to ensure the risk remains as low as possible (39). Risk models such as ours provide a rapid and efficient assessment and should be regularly employed with the most recent information available in order to target monitoring. Between 2018 and 2019, Adams conducted small scale pathogen monitoring in Parque Nacional Volcán Poás, Parque Nacional Tapantí - Macizo Cerro de la Muerte, and Parque Internacional La Amistad, three of the priority areas identified in this paper (39). While all animals sampled in this study were negative for *Bsal*, the sample size was small, roughly 100 animals across multiple sites, only encompassing four salamander species, and the continued monitoring of these at-risk populations is essential. Because anthropogenic activities can and already have played such an important role in the globalization of *Bsal*, *Bd*, *Pd*, and other pathogens, it is crucial to engage stakeholders, i.e. local communities, researchers, ecotourists, and others entering high risk natural areas, in this conservation work. We encourage the development of educational tools designed to inform the public and researchers on the ecological importance of amphibians, their conservation, diseases, and easy actions that can be taken to limit pathogen transmission. Such actions may include responsibly recreating (i.e., limiting recreation to designated trails) and regularly cleaning recreational and/or research equipment, especially when visiting protected areas with a high risk of pathogen introduction. Various studies have demonstrated the efficacy of regulatory signage in natural areas in reducing unwanted behaviors, such as off trail hiking and encroaching upon areas of particular ecological value (8,68–70). Similar studies have additionally shown that such signage is particularly impactful when designed with accessibility and approachability in mind: i.e., visually appealing, non verbose, and written with amicable language (68). Other studies have demonstrated the efficacy of household cleaners, such as 4% bleach, in killing both *Bsal* and *Bd* (71), presenting financially accessible measures for the public to control pathogen spread. Such management initiatives could significantly reduce the risk of pathogen introductions going undetected. Ideally, such initiatives would be developed in collaboration with engaged local communities, which would only further conservation management and education efficacy, helping to control spillover events, and aid in global amphibian conservation (72,73).

**Conclusions and limitations**

*Bsal* is a serious and global threat to salamanders (17–22,74). The pathogen has now been detected in wild amphibians in nine countries and, while it has not yet been detected in the Americas, it is known to be infectious to at least 16 species native to the Americas (75). As our knowledge grows, so does our understanding of which species can be infected by the pathogen and to what degree of morbidity and mortality (76,77). Risk models, such as ours, can be effective tools in developing targeted pathogen monitoring and mitigation planning. In this study, we used ecological niche modeling, salamander diversity, and tourist visitation data in protected natural areas to identify specific locations, in addition to previously identified geographic regions, worthy of monitoring for the emergence of *Bsal*. Compared to Mexico, a much larger proportion of the Costa Rican landmass is predicted to be suitable for *Bsal* (43). We have identified that ~23% of Costa Rica is moderately to highly suitable for *Bsal* and contains a high amount of salamander diversity. It should be noted that species distribution models of species extent and biodiversity can overestimate distribution, thus our models should be interpreted with caution (78). We further pinpointed eight priority regions for surveillance based on the overlapping criteria of *Bsal* ecological suitability, salamander biodiversity, and/or high annual human visitation (Table III, Appendix I). It is our hope that the findings of this study facilitate further pathogen surveillance and prioritized efforts in characterizing the susceptibility of salamander species within high risk areas in Costa Rica. We additionally encourage the development of educational tools in collaboration with local communities designed to inform the public and researchers on amphibian conservation, diseases, and easy steps they can take to limit pathogen transmission, especially when visiting protected areas with high human visitation.

Remote sensing data and ecological niche models are often crucial tools in biodiversity preservation and conservation work, allowing research at an expansive extent with relatively low financial and personnel cost. Working at such a scale entails generalizations and limitations, particularly when the species of interest is significantly small and when complementary data, such as human visitation to natural areas, is limited. Our model does not capture microhabitats, thus future work should be realistic about interpreting this model and cognizantly integrate *in-situ* and local knowledge of the landscape. Additionally, a greater understanding of how people utilize natural areas and move between them would greatly aid in future model building. This risk analysis should supplement existing and future studies and identify novel research questions.

**Acknowledgments**

We would like to thank the Comisión Nacional para la Gestión de la Biodiversidad, specifically Jose Hernandez and Melania Muñoz, for their support and oversight during Henry C. Adams’ graduate studies. We would like to thank Clark Labs, the developers of TerrSet, for making their wonderful software accessible to students. We would like to extend a tremendous thank you to Jeremy Klank, who helped Adams conduct their graduate field research as well as all of our friends and family in Costa Rica and the United States who helped make this research and all the work we do possible with their love and support. Finally, we would like to thank our reviewers, who’s fantastic feedback significantly improved the quality of this manuscript.

**References**

1.	Daszak P, Cunningham AA, Hyatt AD. Emerging Infectious Diseases of Wildlife-- Threats to Biodiversity and Human Health. Science [Internet]. 2000 Jan 21 [cited 2023 Sep 1];287(5452):443–9. Available from: https://www.science.org/doi/full/10.1126/science.287.5452.443

2.	Grant EHC, Muths E, Katz RA, Canessa S, Adams MJ, Ballard JR, et al. Using decision analysis to support proactive management of emerging infectious wildlife diseases. Front Ecol Environ [Internet]. 2017 [cited 2023 Sep 1];15(4):214–21. Available from: https://onlinelibrary.wiley.com/doi/abs/10.1002/fee.1481

3.	Martel A, Vila-Escale M, Fernández-Giberteau D, Martinez-Silvestre A, Canessa S, Van Praet S, et al. Integral chain management of wildlife diseases. Conserv Lett [Internet]. 2020 [cited 2023 Sep 1];13(2):e12707. Available from: https://onlinelibrary.wiley.com/doi/abs/10.1111/conl.12707

4.	Terrestrial Animal Health Code [Internet]. 27th ed. Vol. I General provisions. World Organization for Animal Health (OIE); 2018. Available from: https://www.oie.int/fileadmin/Home/eng/Health_standards/tahc/2018/ en_sommaire.htm

5.	Berger L, Speare R, Daszak P, Green DE, Cunningham AA, Goggin CL, et al. Chytridiomycosis causes amphibian mortality associated with population declines in the rain forests of Australia and Central America. Proc Natl Acad Sci [Internet]. 1998 Jul 21 [cited 2023 Sep 1];95(15):9031–6. Available from: https://www.pnas.org/doi/abs/10.1073/pnas.95.15.9031

6.	Scheele BC, Pasmans F, Skerratt LF, Berger L, Martel A, Beukema W, et al. Amphibian fungal panzootic causes catastrophic and ongoing loss of biodiversity. Science [Internet]. 2019 Mar 29 [cited 2023 Sep 1];363(6434):1459–63. Available from: https://www.science.org/doi/full/10.1126/science.aav0379

7.	O’Hanlon SJ, Rieux A, Farrer RA, Rosa GM, Waldman B, Bataille A, et al. Recent Asian origin of chytrid fungi causing global amphibian declines. Science [Internet]. 2018 May 11 [cited 2023 Sep 1];360(6389):621–7. Available from: https://www.science.org/doi/10.1126/science.aar1965

8.	Crump ML, Hensley FR, Clark KL. Apparent Decline of the Golden Toad: Underground or Extinct? Copeia [Internet]. 1992 [cited 2023 Sep 1];1992(2):413–20. Available from: https://www.jstor.org/stable/1446201

9.	Puschendorf R, Carnaval AC, VanDerWal J, Zumbado-Ulate H, Chaves G, Bolaños F, et al. Distribution models for the amphibian chytrid Batrachochytrium dendrobatidis in Costa Rica: proposing climatic refuges as a conservation tool. Divers Distrib [Internet]. 2009 [cited 2023 Sep 1];15(3):401–8. Available from: https://onlinelibrary.wiley.com/doi/abs/10.1111/j.1472-4642.2008.00548.x

10.	Miller CA, Taboue GCT, Ekane MMP, Robak M, Clee PRS, Richards-Zawacki C, et al. Distribution modeling and lineage diversity of the chytrid fungus Batrachochytrium dendrobatidis (Bd) in a central African amphibian hotspot. PLOS ONE [Internet]. 2018 Jun 20 [cited 2023 Sep 1];13(6):e0199288. Available from: https://journals.plos.org/plosone/article?id=10.1371/journal.pone.0199288

11.	Voyles J, Kilpatrick AM, Collins JP, Fisher MC, Frick WF, McCallum H, et al. Moving Beyond Too Little, Too Late: Managing Emerging Infectious Diseases in Wild Populations Requires International Policy and Partnerships. EcoHealth [Internet]. 2015 Sep 1 [cited 2023 Sep 1];12(3):404–7. Available from: https://doi.org/10.1007/s10393-014-0980-5

12.	Blehert DS, Hicks AC, Behr M, Meteyer CU, Berlowski-Zier BM, Buckles EL, et al. Bat White-Nose Syndrome: An Emerging Fungal Pathogen? Science [Internet]. 2009 Jan 9 [cited 2023 Sep 1];323(5911):227–227. Available from: https://www.science.org/doi/full/10.1126/science.1163874

13.	The National Plan for Assisting States, Tribes and Federal Agencies in Managing White-Nose Syndrome in Bats [Internet]. Arlington, VA: U.S. Fish and Wildlife Service; 2011 p. 17. Available from: https://pubs.usgs.gov/publication/70039214ional_plan_may_2011.pdf

14.	Bernard RF, Reichard JD, Coleman JTH, Blackwood JC, Verant ML, Segers JL, et al. Identifying research needs to inform white-nose syndrome management decisions. Conserv Sci Pract [Internet]. 2020 [cited 2023 Sep 1];2(8):e220. Available from: https://onlinelibrary.wiley.com/doi/abs/10.1111/csp2.220

15.	Hicks LL, Schwab NA, Homyack JA, Jones JE, Maxell BA, Burkholder BO. A statistical approach to white-nose syndrome surveillance monitoring using acoustic data. PLOS ONE [Internet]. 2020 Oct 22 [cited 2023 Sep 1];15(10):e0241052. Available from: https://journals.plos.org/plosone/article?id=10.1371/journal.pone.0241052

16.	Gabriel KT, McDonald AG, Lutsch KE, Pattavina PE, Morris KM, Ferrall EA, et al. Development of a multi-year white-nose syndrome mitigation strategy using antifungal volatile organic compounds. PLOS ONE [Internet]. 2022 Dec 1 [cited 2023 Sep 1];17(12):e0278603. Available from: https://journals.plos.org/plosone/article?id=10.1371/journal.pone.0278603

17.	Martel A, Spitzen-van der Sluijs A, Blooi M, Bert W, Ducatelle R, Fisher MC, et al. Batrachochytrium salamandrivorans sp. nov. causes lethal chytridiomycosis in amphibians. Proc Natl Acad Sci [Internet]. 2013 Sep 17 [cited 2023 Sep 1];110(38):15325–9. Available from: https://www.pnas.org/doi/abs/10.1073/pnas.1307356110

18.	Martel A, Blooi M, Adriaensen C, Van Rooij P, Beukema W, Fisher MC, et al. Recent introduction of a chytrid fungus endangers Western Palearctic salamanders. Science [Internet]. 2014 Oct 31 [cited 2023 Sep 1];346(6209):630–1. Available from: https://www.science.org/doi/full/10.1126/science.1258268

19.	Stegen G, Pasmans F, Schmidt BR, Rouffaer LO, Van Praet S, Schaub M, et al. Drivers of salamander extirpation mediated by Batrachochytrium salamandrivorans. Nature [Internet]. 2017 Apr [cited 2023 Sep 1];544(7650):353–6. Available from: https://www.nature.com/articles/nature22059

20.	Carter ED, Miller DL, Peterson AC, Sutton WB, Cusaac JPW, Spatz JA, et al. Conservation risk of Batrachochytrium salamandrivorans to endemic lungless salamanders. Conserv Lett [Internet]. 2020 [cited 2023 Sep 1];13(1):e12675. Available from: https://onlinelibrary.wiley.com/doi/abs/10.1111/conl.12675

21.	Lötters S, Veith M, Wagner N, Martel A, Pasmans F. Bsal-driven salamander mortality pre-dates the European index outbreak. Salamandra. 2020;56(3):239–42.

22.	Lötters S, Wagner N, Albaladejo G, Böning P, Dalbeck L, Düssel-Siebert H, et al. The amphibian pathogen Batrachochytrium salamandrivorans in the hotspot of its European invasive range: past – present – future. Salamandra. 2020 Aug 15;56:173–88.

23.	Laking AE, Ngo HN, Pasmans F, Martel A, Nguyen TT. Batrachochytrium salamandrivorans is the predominant chytrid fungus in Vietnamese salamanders. Sci Rep [Internet]. 2017 Mar 13 [cited 2023 Sep 1];7(1):44443. Available from: https://www.nature.com/articles/srep44443

24.	Cunningham AA, Beckmann K, Perkins M, Fitzpatrick L, Cromie R, Redbond J, et al. Emerging disease in UK amphibians. Vet Rec [Internet]. 2015 May 2 [cited 2023 Sep 1];176(18):468. Available from: https://www.proquest.com/docview/1779604632/abstract/AEE3EB2FE60545E5PQ/1

25.	Nguyen TT, Nguyen TV, Ziegler T, Pasmans F, Martel A. Trade in wild anurans vectors the urodelan pathogen Batrachochytrium salamandrivorans into Europe. Amphib-Reptil [Internet]. 2017 Jan 1 [cited 2023 Sep 1];38(4):554–6. Available from: https://brill.com/view/journals/amre/38/4/article-p554_14.xml

26.	Towe AE, Gray MJ, Carter ED, Wilber MQ, Ossiboff RJ, Ash K, et al. Batrachochytrium salamandrivorans Can Devour More than Salamanders. J Wildl Dis. 2021 Oct 1;57(4):942–8.

27.	Basanta MD, Avila-Akerberg V, Byrne AQ, Castellanos-Morales G, Martínez TMG, Maldonado-López Y, et al. The fungal pathogen Batrachochytrium salamandrivorans is not detected in wild and captive amphibians from Mexico. 2022 May 6 [cited 2024 Jul 15]; Available from: https://peerj.com/articles/14117

28.	Salamander chytrid fungus (Batrachochytrium salamandrivorans) in the United States—Developing research, monitoring, and management strategies. [Internet]. Reston, VA: U.S. Geological Survey; 2016 p. 16. Report No.: 2015–1233. Available from: https://doi.org/10.3133/ofr20151233

29.	Gray MJ, Lewis JP, Nanjappa P, Klocke B, Pasmans F, Martel A, et al. Batrachochytrium salamandrivorans: The North American Response and a Call for Action. PLOS Pathog [Internet]. 2015 Dec 10 [cited 2023 Sep 1];11(12):e1005251. Available from: https://journals.plos.org/plospathogens/article?id=10.1371/journal.ppat.1005251

30.	Richgels KLD, Russell RE, Adams MJ, White CL, Grant EHC. Spatial variation in risk and consequence of *Batrachochytrium salamandrivorans* introduction in the USA. R Soc Open Sci [Internet]. 2016 Feb [cited 2023 Sep 1];3(2):150616. Available from: https://royalsocietypublishing.org/doi/10.1098/rsos.150616

31.	Spitzen-van der Sluijs A, Martel A, Asselberghs J, Bales EK, Beukema W, Bletz MC, et al. Expanding Distribution of Lethal Amphibian Fungus Batrachochytrium salamandrivorans in Europe. Emerg Infect Dis [Internet]. 2016 Jul [cited 2023 Sep 1];22(7):1286–8. Available from: https://www.ncbi.nlm.nih.gov/pmc/articles/PMC4918153/

32.	Govindarajulu P, Matthews E, Ovaska K. Swabbing for Batrachochytrium salamandrivorans on wild Rough-skinned Newts (Taricha granulosa) and pet-traded amphibians on Southern Vancouver Island. Herpetol Rev. 2017;48:564–8.

33.	Klocke B, Becker M, Lewis J, Fleischer RC, Muletz-Wolz CR, Rockwood L, et al. Batrachochytrium salamandrivorans not detected in U.S. survey of pet salamanders. Sci Rep [Internet]. 2017 Oct 13 [cited 2023 Sep 1];7(1):13132. Available from: https://www.nature.com/articles/s41598-017-13500-2

34.	Malagon DA, Melara LA, Prosper OF, Lenhart S, Carter ED, Fordyce JA, et al. Host density and habitat structure influence host contact rates and Batrachochytrium salamandrivorans transmission. Sci Rep [Internet]. 2020 Mar 27 [cited 2023 Sep 5];10(1):5584. Available from: https://www.nature.com/articles/s41598-020-62351-x

35.	Tompros A, Dean AD, Fenton A, Wilber MQ, Carter ED, Gray MJ. Frequency-dependent transmission of Batrachochytrium salamandrivorans in eastern newts. Transbound Emerg Dis [Internet]. 2022 [cited 2023 Sep 5];69(2):731–41. Available from: https://onlinelibrary.wiley.com/doi/abs/10.1111/tbed.14043

36.	Yap TA, Koo MS, Ambrose RF, Wake DB, Vredenburg VT. Averting a North American biodiversity crisis. Science [Internet]. 2015 Jul 31 [cited 2023 Sep 1];349(6247):481–2. Available from: https://www.science.org/doi/full/10.1126/science.aab1052

37.	Parrott JC, Shepack A, Burkart D, LaBumbard B, Scimè P, Baruch E, et al. Survey of Pathogenic Chytrid Fungi (Batrachochytrium dendrobatidis and B. salamandrivorans) in Salamanders from Three Mountain Ranges in Europe and the Americas. EcoHealth [Internet]. 2017 Jun 1 [cited 2023 Sep 1];14(2):296–302. Available from: https://doi.org/10.1007/s10393-016-1188-7

38.	Savage JM. The Amphibians and Reptiles of Costa Rica: A Herpetofauna Between Two Continents, Between Two Seas. University of Chicago Press; 2002. 1076 p.

39.	Adams HC. Assessing the Emergence of a Fungal Pathogen (Batrachochytrium salamandrivorans) for the Conservation of Costa Rican Salamanders [Internet] [M.S.]. [Athens, GA]: University of Georgia; 2020 [cited 2023 Sep 1]. Available from: https://www.proquest.com/docview/2446712399/abstract/C20F3CCB48DC4107PQ/1

40.	Anuario Estadístico de Turismo 2018. San José, Costa Rica: Instituto Costarricense de Turismo; 2018.

41.	Cheng TL, Rovito SM, Wake DB, Vredenburg VT. Coincident mass extirpation of neotropical amphibians with the emergence of the infectious fungal pathogen Batrachochytrium dendrobatidis. Proc Natl Acad Sci [Internet]. 2011 Jun 7 [cited 2023 Sep 1];108(23):9502–7. Available from: https://www.pnas.org/doi/10.1073/pnas.1105538108

42.	Katz TS, Zellmer AJ. Comparison of model selection technique performance in predicting the spread of newly invasive species: a case study with Batrachochytrium salamandrivorans. Biol Invasions. 2018;20:2107–19.

43.	Basanta MD, Rebollar EA, Parra-Olea G. Potential risk of Batrachochytrium salamandrivorans in Mexico. PLOS ONE [Internet]. 2019 Feb 12 [cited 2023 Sep 1];14(2):e0211960. Available from: https://journals.plos.org/plosone/article?id=10.1371/journal.pone.0211960

44.	García-Rodríguez A, Basanta MD, García-Castillo MG, Zumbado-Ulate H, Neam K, Rovito S, et al. Anticipating the potential impacts of Batrachochytrium salamandrivorans on Neotropical salamander diversity. Biotropica [Internet]. 2022 [cited 2023 Sep 1];54(1):157–69. Available from: https://onlinelibrary.wiley.com/doi/abs/10.1111/btp.13042

45.	Beukema W, Erens J, Schulz V, Stegen G, Spitzen-van der Sluijs A, Stark T, et al. Landscape epidemiology of Batrachochytrium salamandrivorans: reconciling data limitations and conservation urgency. Ecol Appl [Internet]. 2021 [cited 2023 Sep 1];31(5):e02342. Available from: https://onlinelibrary.wiley.com/doi/abs/10.1002/eap.2342

46.	Rodríguez-Castañeda G, Hof AR, Jansson R, Harding LE. Predicting the Fate of Biodiversity Using Species’ Distribution Models: Enhancing Model Comparability and Repeatability. PLOS ONE [Internet]. 2012 Sep 11 [cited 2023 Sep 1];7(9):e44402. Available from: https://journals.plos.org/plosone/article?id=10.1371/journal.pone.0044402

47.	Konowalik K, Nosol A. Evaluation metrics and validation of presence-only species distribution models based on distributional maps with varying coverage. Sci Rep [Internet]. 2021 Jan 15 [cited 2023 Sep 1];11(1):1482. Available from: https://www.nature.com/articles/s41598-020-80062-1

48.	Yuan Z, Martel A, Wu J, Van Praet S, Canessa S, Pasmans F. Widespread occurrence of an emerging fungal pathogen in heavily traded Chinese urodelan species. Conserv Lett [Internet]. 2018 [cited 2023 Sep 1];11(4):e12436. Available from: https://onlinelibrary.wiley.com/doi/abs/10.1111/conl.12436

49.	Clark Labs. Terrset. Worcester, MA: Clark Labs; 2017.

50.	ANURA (spatial data) [Internet]. The IUCN Red List of Threatened Species.; 2017 [cited 2020 Feb 2]. Available from: www.iucnredlist.org

51.	IUCN (The International Union for Conservation of Nature). CAUDATA (spatial data) [Internet]. The IUCN Red List of Threatened Species; 2017 [cited 2020 Feb 2]. Available from: www.iucnredlist.org

52.	Guisan A, Zimmermann NE. Predictive habitat distribution models in ecology. Ecol Model [Internet]. 2000 Dec 5 [cited 2023 Sep 1];135(2):147–86. Available from: https://www.sciencedirect.com/science/article/pii/S0304380000003549

53.	Elith* J, H. Graham* C, P. Anderson R, Dudík M, Ferrier S, Guisan A, et al. Novel methods improve prediction of species’ distributions from occurrence data. Ecography [Internet]. 2006 [cited 2023 Sep 1];29(2):129–51. Available from: https://onlinelibrary.wiley.com/doi/abs/10.1111/j.2006.0906-7590.04596.x

54.	Pearson RG, Raxworthy C, Nakamura M, Townsend Peterson A. Predicting species distributions from small numbers of occurrence records: a test case using cryptic geckos in Madagascar. J Biogeogr [Internet]. 2007 [cited 2023 Sep 1];34(1):102–17. Available from: https://onlinelibrary.wiley.com/doi/abs/10.1111/j.1365-2699.2006.01594.x

55.	Beukema W, Martel A, Nguyen TT, Goka K, Schmeller DS, Yuan Z, et al. Environmental context and differences between native and invasive observed niches of Batrachochytrium salamandrivorans affect invasion risk assessments in the Western Palaearctic. Divers Distrib [Internet]. 2018 [cited 2023 Sep 1];24(12):1788–801. Available from: https://onlinelibrary.wiley.com/doi/abs/10.1111/ddi.12795

56.	Hijmans RJ, Cameron SE, Parra JL, Jones PG, Jarvis A. Very high resolution interpolated climate surfaces for global land areas. Int J Climatol [Internet]. 2005 [cited 2023 Sep 1];25(15):1965–78. Available from: https://onlinelibrary.wiley.com/doi/abs/10.1002/joc.1276

57.	Elith J, Phillips SJ, Hastie T, Dudík M, Chee YE, Yates CJ. A statistical explanation of MaxEnt for ecologists: Statistical explanation of MaxEnt. Divers Distrib. 2011 Jan;17(1):43–57.

58.	Buckley LB, Jetz W. Environmental and historical constraints on global patterns of amphibian richness. Proc Biol Sci. 2007 May 7;274(1614):1167–73.

59.	Carter ED, Bletz MC, Sage ML, LaBumbard B, Rollins-Smith LA, Woodhams DC, et al. Winter is coming–Temperature affects immune defenses and susceptibility to Batrachochytrium salamandrivorans. PLOS Pathog [Internet]. 2021 Feb 18 [cited 2024 Feb 5];17(2):e1009234. Available from: https://journals.plos.org/plospathogens/article?id=10.1371/journal.ppat.1009234

60.	Protected Planet: The World Database on Protected Areas (WDPA) [Internet]. Cambridge, UK: UNEP-WCMC and IUCN.: UNEP-WCMC and IUCN; 2020. Available from: www.protectedplanet.net

61.	Scheffers BR, Evans TA, Williams SE, Edwards DP. Microhabitats in the tropics buffer temperature in a globally coherent manner. Biol Lett [Internet]. 2014 Dec [cited 2023 Sep 1];10(12):20140819. Available from: https://royalsocietypublishing.org/doi/full/10.1098/rsbl.2014.0819

62.	Scheffers BR, Edwards DP, Diesmos A, Williams SE, Evans TA. Microhabitats reduce animal’s exposure to climate extremes. Glob Change Biol [Internet]. 2014 [cited 2023 Sep 1];20(2):495–503. Available from: https://onlinelibrary.wiley.com/doi/abs/10.1111/gcb.12439

63.	Schmidt BR, Bozzuto C, Lötters S, Steinfartz S. Dynamics of host populations affected by the emerging fungal pathogen Batrachochytrium salamandrivorans. R Soc Open Sci. 2017;4(3):160801.

64.	Ponce M, Navarro D, Morales Flores R, Batista A. A new salamander of the genus Bolitoglossa (Caudata: Plethodontidae) from the highlands of western Panama. Zootaxa. 2022 Apr 29;5129.

65.	DiRenzo GV, Longo AV, Muletz-Wolz CR, Pessier AP, Goodheart JA, Lips KR. Plethodontid salamanders show variable disease dynamics in response to Batrachochytrium salamandrivorans chytridiomycosis. Biol Invasions [Internet]. 2021 Sep 1 [cited 2023 Sep 1];23(9):2797–815. Available from: https://doi.org/10.1007/s10530-021-02536-1

66.	Lips KR, Mendelson III JR, Muñoz-Alonso A, Canseco-Márquez L, Mulcahy DG. Amphibian population declines in montane southern Mexico: resurveys of historical localities. Biol Conserv [Internet]. 2004 Oct 1 [cited 2023 Sep 11];119(4):555–64. Available from: https://www.sciencedirect.com/science/article/pii/S0006320704000217

67.	Longo AV, Fleischer RC, Lips KR. Double trouble: co-infections of chytrid fungi will severely impact widely distributed newts. Biol Invasions [Internet]. 2019 Jun 1 [cited 2023 Sep 1];21(6):2233–45. Available from: https://doi.org/10.1007/s10530-019-01973-3

68.	Scane S. Evaluating the Effectiveness of Signage in Conservation Areas, Natural Areas and Zoos to Enhance the Education of Eco-tourists. In 2020 [cited 2023 Sep 11]. Available from: https://www.semanticscholar.org/paper/Evaluating-the-Effectiveness-of-Signage-in-Areas%2C-Scane/3f14bfc7c10a09f6522435dcce6b63ea168f3d55

69.	Allbrook DL, Quinn JL. The effectiveness of regulatory signs in controlling human behaviour and Northern gannet (Morus bassanus) disturbance during breeding: an experimental test. J Nat Conserv. 2020 Dec;58:125915.

70.	Hockett KS, Marion JL, Leung YF. The efficacy of combined educational and site management actions in reducing off-trail hiking in an urban-proximate protected area. J Environ Manage [Internet]. 2017 Dec 1 [cited 2023 Sep 1];203:17–28. Available from: https://www.sciencedirect.com/science/article/pii/S0301479717306643

71.	Van Rooij P, Pasmans F, Coen Y, Martel A. Efficacy of chemical disinfectants for the containment of the salamander chytrid fungus Batrachochytrium salamandrivorans. PLOS ONE [Internet]. 2017 Oct 12 [cited 2023 Sep 1];12(10):e0186269. Available from: https://journals.plos.org/plosone/article?id=10.1371/journal.pone.0186269

72.	Barnes M, Schmitz P. Community engagement matters (now more than ever). Stanf Soc Innov Rev. 2016;14(2):32–9.

73.	Nyirenda D, Gooding K, Sambakunsi R, Seyama L, Mfutso-Bengo J, Manda Taylor L, et al. Strengthening ethical community engagement in contemporary Malawi. Wellcome Open Res [Internet]. 2019 Mar 18 [cited 2023 Sep 20];3:115. Available from: https://www.ncbi.nlm.nih.gov/pmc/articles/PMC6259484/

74.	Gray MJ, Carter ED, Piovia-Scott J, Cusaac JPW, Peterson AC, Whetstone RD, et al. Broad host susceptibility of North American amphibian species to Batrachochytrium salamandrivorans suggests high invasion potential and biodiversity risk. Nat Commun [Internet]. 2023 Jun 5 [cited 2023 Sep 5];14(1):3270. Available from: https://www.nature.com/articles/s41467-023-38979-4

75.	Castro Monzon F, Rödel MO, Ruland F, Parra-Olea G, Jeschke JM. Batrachochytrium salamandrivorans’ Amphibian Host Species and Invasion Range. EcoHealth [Internet]. 2022 Dec 1 [cited 2023 Sep 5];19(4):475–86. Available from: https://doi.org/10.1007/s10393-022-01620-9

76.	Li Z, Verbrugghe E, Konstanjšek R, Lukač M, Pasmans F, Cizelj I, et al. Dampened virulence and limited proliferation of Batrachochytrium salamandrivorans during subclinical infection of the troglobiont olm (Proteus anguinus). Sci Rep [Internet]. 2020 Oct 5 [cited 2023 Sep 5];10(1):16480. Available from: https://www.nature.com/articles/s41598-020-73800-y

77.	Schulz V, Schulz A, Klamke M, Preissler K, Sabino-Pinto J, Müsken M, et al. Batrachochytrium salamandrivorans in the ruhr district, germany: History, distribution, decline dynamics and disease symptoms of the salamander plague. 56 [Internet]. 2020 Aug 15 [cited 2023 Sep 5]; Available from: https://repository.helmholtz-hzi.de/handle/10033/622523

78.	Graham CH, Hijmans RJ. A comparison of methods for mapping species ranges and species richness. Glob Ecol Biogeogr [Internet]. 2006 [cited 2024 Jul 17];15(6):578–87. Available from: https://onlinelibrary.wiley.com/doi/abs/10.1111/j.1466-8238.2006.00257.x

**Supporting Information Captions**

**S1 Appendix Table I. Human visitation rate per annum for Costa Rican protected areas in 2018 (1).** Note, visitation data was not found for all protected areas. Visitation numbers include both international and local visitors.

**S1 Appendix Table II. Salamander species, total land area, and protected land area in areas of high, medium, and low predicted *Bsal* suitability.**

**S1 Appendix Table III. Priority regions statistics.** This table lists the eight high priority areas suggested for monitoring. For each priority region, the total area in square kilometers that is considered highly and moderately suitable for *Bsal* is given. The average and range of suitability are listed (one indicates the habitat is highly suitable and zero indicates the habitat is completely unsuitable). Park visitation numbers are from 2018.

**S1 Appendix Figure I. Salamander gamma diversity in protected and non-protected areas.** Darker shades indicate higher gamma diversity (greater number of endemic species compared to all of Costa Rica). Protected areas are outlined and the number of visitors in 2018 is indicated by pattern.

**S1 Appendix Table IV. Pearson’s correlation values for bioclimatic variables included in the selected model.** Cells with correlation values greater than an absolute value of 0.7 have been shaded with darker shading indicating greater correlation.

**S1 Appendix Table V. Analysis of variable contributions in the selected model.** To determine percent contribution, in each iteration of the training algorithm, the increase in regularized gain is added to the contribution of the corresponding variable or subtracted from it if the change to the absolute value of lambda is negative. For permutation importance, for each environmental variable in turn, the values of that variable on training presence and background data are randomly permuted. The model is reevaluated on the permuted data, and the resulting drop in training AUC is shown in the table, normalized to percentages. Variable contributions should be interpreted with caution when the predictor variables are correlated.

**S1 Appendix Figure II. Jackknife test of variable importance for the selected model.** Displays the variable importance of each environmental variable used in the selected model. Variable bioclimatic 2 contributes most to the model on its own.

**S1 Appendix Figure III. Analysis of omission/commission for the selected model.** Displays the omission rate, as calculated on the training presence records, and predicted area as a function of the cumulative threshold.

**S1 Appendix Figure IV. The Receiver Operating Characteristic (ROC) curve for the selected model.**

**S1 Appendix Table VI. Pearson’s correlation values for bioclimatic variables included in the all variables model.** Cells with correlation values greater than an absolute value of 0.7 have been shaded with darker shading indicating greater correlation.

**S1 Appendix Table VII. Analysis of variable contributions for the all variables model.** To determine percent contribution, in each iteration of the training algorithm, the increase in regularized gain is added to the contribution of the corresponding variable or subtracted from it if the change to the absolute value of lambda is negative. For permutation importance, for each environmental variable in turn, the values of that variable on training presence and background data are randomly permuted. The model is reevaluated on the permuted data, and the resulting drop in training AUC is shown in the table, normalized to percentages. Variable contributions should be interpreted with caution when the predictor variables are correlated.

**S1 Appendix Figure V. Jackknife test of variable importance for the all variables model.** Displays the variable importance of each environmental variable used in the all variables model. Variable bioclimatic 2 contributes most to the model on its own.

**S1 Appendix Figure VI. Analysis of omission/commission for the all variables model.** Displays the omission rate, as calculated on the training presence records, and predicted area as a function of the cumulative threshold.

**S1 Appendix Figure VII. The Receiver Operating Characteristic (ROC) curve for the all variables model.**

**S1 Appendix Figure VIII. Predicted *Bsal* distribution in Costa Rica using the all variables model.** Warmer colors indicate higher suitability and cooler colors indicate lower suitability. Suitability ranges from 0-1. For more information on suitability, see full manuscript.

**S1 Appendix Figure IX. Sensitivity. Difference between the selected model (Figure 4 in manuscript) and the all variables model (Figure VII).** Created by subtracting the suitability values of the selected model from the suitability values of the all variables model, which used uncorrelated variables. Positive values indicate greater suitability in the model chosen for the manuscript. Negative values indicate lower suitability in the model chosen for the manuscript. For more information on suitability, see the full manuscript.

**S1 Appendix Table VIII. Pearson’s correlation values for bioclimatic variables included in the limited model.**

**S1 Appendix Table IX. Analysis of variable contributions in the limited model.**

**S1 Appendix Figure X. Jackknife test of variable importance for the limited model.** Displays the variable importance of each environmental variable used in the limited model. Variable bioclimatic 2 contributes most to the model on its own.

**S1 Appendix Figure XI. Analysis of omission/commission for the limited model.** Displays the omission rate, as calculated on the training presence records, and predicted area as a function of the cumulative threshold.

**S1 Appendix Figure XII. The Receiver Operating Characteristic (ROC) curve for the limited model.**

**S1 Appendix Figure XIII. Predicted *Bsal* distribution in Costa Rica using the limited model.** Warmer colors indicate higher suitability and cooler colors indicate lower suitability. Suitability ranges from 0-1. For more information on suitability, see full manuscript.

**S1 Appendix Figure XIV. Sensitivity. Difference between the selected model (Figure 4 in manuscript) and the limited model, which used select, uncorrelated bioclimatic variables (Figure XII).** Created by subtracting the suitability values of the selected model from the suitability values of the limited model that used uncorrelated variables. Positive values indicate greater suitability in the model chosen for the manuscript. Negative values indicate lower suitability in the model chosen for the manuscript. For more information on suitability, see the full manuscript.
